# Unraveling CD8 lineage decisions reveals that functionally distinct CD8^+^ T cells are selected by different MHC-I thymic peptides

**DOI:** 10.1038/s41590-025-02411-4

**Published:** 2026-01-19

**Authors:** Miho Shinzawa, Nicole Ramos, Khanh Bui, William Hajjar, Assiatu Crossman, Xiongfong Chen, Margaret Cam, Yousuke Takahama, Alfred Singer

**Affiliations:** 1 Experimental Immunology Branch, National Cancer Institute, National Institutes of Health, Bethesda, MD USA; 2 CCR Collaborative Bioinformatics Resource, Office of Science and Technology Resources, Office of Director, Center for Cancer Research, National Cancer Institute, National Institutes of Health, Bethesda, MD USA; 3 CCR-SF Bioinformatics Group, Advanced Biomedical Computational Science, Biomedical Informatics and Data Science Directorate, Frederick National Laboratory for Cancer Research, Frederick, MD USA; 4 Thymus Biology Section, Experimental Immunology Branch, National Cancer Institute, National Institutes of Health, Bethesda, MD USA

**Keywords:** CD8-positive T cells, Lymphocyte differentiation

## Abstract

Thymocytes signaled by T cell antigen receptors to undergo positive selection acquire different functional fates while migrating through the thymus, but how this occurs remains uncertain. We now report that encoding CD8 co-receptors in both *Cd4* and *Cd8* gene loci modulates major histocompatibility complex (MHC-I) class I T cell antigen receptor signaling duration to generate all potential CD8^+^ T cell subsets. Strikingly, such mice revealed that functionally different CD8^+^ T cells are selected by different MHC-I thymic peptides. Thymocytes signaled by β5t-peptides produced by thymoproteasomes exclusively expressed in the thymic cortex invariably become cytotoxic CD8^+^ T cells indicating their signaling ceases when thymocytes leave the cortex; whereas thymocytes signaled by nonβ5t-peptides expressed throughout the thymus become either helper or innate memory CD8^+^ T cells because their signaling persists or recurs outside the cortex. Thus, it is because of their different thymic distributions that different MHC-I peptides select functionally different CD8^+^ T cells, integrating peptide specificity and CD8^+^ T cell function during positive selection and thymocyte migration.

## Abstract (web summary)

Shinzawa et al. developed CD8^Dual^ mice that express CD8 co-receptors from both *Cd4* and *Cd8* gene loci. Using this model they find that thymic distribution of major histocompatibility complex class I peptides influences lineage commitment and CD8^+^ T cell function.

## Main

During T cell development in the thymus, immature thymocytes are signaled by T cell antigen receptors (TCRs) to undergo positive selection and to differentiate into CD4^+^ or CD8^+^ mature T cells that possess either helper or cytotoxic function^1–3^. Thymocytes that are induced to express the helper factor ThPOK differentiate into helper T cells and those that are induced to express the cytotoxic factor Runx3d differentiate into cytotoxic T cells^4–8^. The lineage factor that TCR-signaled thymocytes express depends on the ligand specificity of their TCR in that TCR–CD8 complexes that engage MHC-I ligands signal thymocytes to become cytotoxic T cells, whereas TCR–CD4 complexes that engage MHC-II ligands signal thymocytes to become helper T cells^9–11^. How TCR/co-receptor signaling induces thymocytes to express one or the other lineage factor during positive selection remains controversial and incompletely understood.

T cell lineage fate determination in the thymus is currently best described by the kinetic signaling model, which is based on the fact that TCR signaling of immature CD4^+^CD8^+^ double-positive thymocytes initially terminates *Cd8* gene expression, regardless of TCR’s ligand specificity^12,13^. When *Cd8* gene expression is terminated, TCR signaling dependent on *Cd8*-encoded co-receptors becomes disrupted, whereas TCR signaling that is independent of *Cd8*-encoded co-receptors persists^12–15^. Consequently, the key concept of kinetic signaling is that T cell lineage fate is determined during positive selection by whether TCR signaling is persistent or disrupted: TCR signaling persistence induces ThPOK, which directs thymocyte differentiation into helper T cells^16,17^, whereas TCR signaling disruption allows thymic cytokines to induce Runx3d, which directs thymocyte differentiation into cytotoxic T cells^18,19^. Interestingly, six different thymic cytokines (interleukin (IL)-7, IL-15, IL-6, interferon gamma (IFNγ), thymic stromal lymphopoietin (TSLP) and transforming growth factor-beta (TGFβ)) have been identified as inducing Runx3d expression and CD8^+^ T cell differentiation when TCR signaling is disrupted during positive selection^19^.

Understanding of T cell lineage fate determination was substantially advanced by a recent study using ‘FlipFlop’ mice in which CD4 and CD8 co-receptors were encoded in opposite *Cd4* and *Cd8* gene loci^20^. Remarkably, FlipFlop mice generated a functionally reversed T cell immune system in which CD8^+^/MHC-I T cells are helpers and CD4^+^/MHC-II T cells are cytotoxic effectors^20^. Thus, this study revealed that T cell lineage fate is not determined by CD4/CD8 co-receptors themselves, but is instead determined by *Cd4* and *Cd8* gene loci that encode CD4/CD8 co-receptors and regulate the kinetics of co-receptor-dependent TCR signaling during positive selection^13,20,21^. However, the possibility that factors other than co-receptor gene loci might also affect T cell lineage fate determination has not been addressed and remains unknown.

The present study was undertaken to specifically identify mechanisms underlying CD8^+^ T cell lineage fate determination during MHC-I positive selection in the thymus. To do so, we constructed CD8^Dual^ mice, which encode CD8 co-receptors in both *Cd4* and *Cd8* gene loci to focus specifically on functional lineage fate decisions by MHC-I TCR-signaled thymocytes. We found that CD8^Dual^ mice are unique in generating both helper and cytotoxic CD8^+^ T cells, and this made it possible to discover that functionally distinct CD8^+^ T cell lineage fates are induced by different MHC-I thymic selecting peptides. Surprisingly, we found that MHC-I thymic selecting peptides expressed exclusively in the thymic cortex only stimulate generation of cytotoxic CD8^+^ T cells indicating that TCR signaling ceases when thymocytes disengage from the cortex, whereas MHC-I thymic selecting peptides that are expressed throughout the thymus stimulate generation of helper and innate memory (IM) CD8^+^ T cells indicating that their TCR signaling continues or recurs outside the cortex. We conclude that it is because MHC-I TCR signaling persistence/disruption dictates CD8^+^ T cell lineage fates that different MHC-I thymic peptides select functionally distinct (helper, cytotoxic and IM) CD8^+^ T cell subsets. Thus, this study integrates peptide specificity, T cell function and thymic migration to indicate how developing CD8^+^ T cells acquire different functionalities during MHC-I positive selection in the thymus.

## Results

### Mice with *Cd4* and *Cd8* gene loci that both encode CD8 co-receptors

**Fig. 1:**
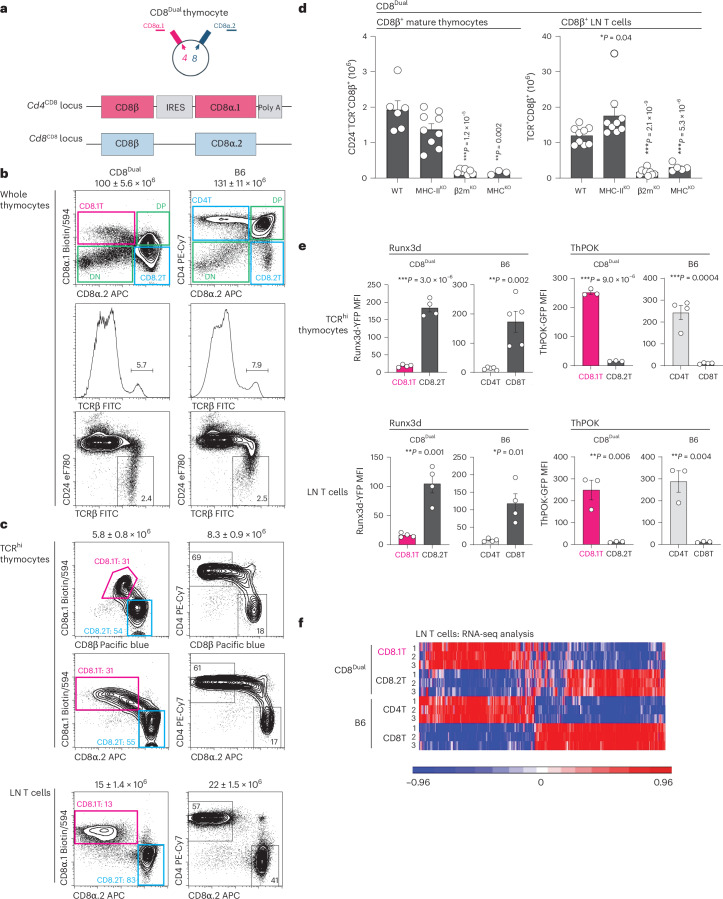
Characterization of CD8^Dual^ mice. **a**, Top: schematic of double-positive thymocytes from CD8^Dual^ mice. The altered *Cd4* gene locus encodes CD8α.1 co-receptors (composed of CD8α.1CD8β dimers), and the endogenous *Cd8* gene locus encodes CD8α.2 co-receptors (composed of CD8α.2CD8β dimers). Bottom: schematic of the altered *Cd4*^CD8^ and endogenous *Cd8*^CD8^ genes in CD8^Dual^ mice; IRES, internal ribosome entry site; poly A, polyadenylation signals. **b**, Flow cytometry analysis of whole thymocytes from CD8^Dual^ (*n* = 16) and B6 (*n* = 15) mice (representative of 15 independent experiments). Top: *Cd4*-encoded co-receptors (CD8α.1 or CD4) versus *Cd8*-encoded co-receptor CD8α.2 profile, showing four thymocyte subsets; double-negative (DN), double-positive (DP) and two single-positive T cell subsets. Total cell number (mean ± s.e.m) is shown above profiles. Middle: TCRβ histogram, showing TCRβ^hi^ (TCR^hi^) thymocytes. Bottom: CD24 versus TCRβ profile, showing CD24^−^TCR^+^ mature thymocytes. **c**, Flow cytometry analysis of TCR^hi^ thymocytes and TCR^+^ LN T cells from CD8^Dual^ (*n* = 16) and B6 (*n* = 15) mice, showing *Cd4*-encoded co-receptor (CD8α.1 or CD4) versus *Cd8*-encoded co-receptor (CD8α.2) or CD8β profiles. TCR expression of LN cells are shown in Extended Data Fig. 1b. Numbers (mean ± s.e.m.) of TCR^hi^ thymocytes and LN T cells are shown above profiles (representative of 15 independent experiments). **d**, Numbers of CD8β^+^ T cells among CD24^−^TCR^+^ mature thymocytes (T) and TCR^+^ LN T cells (L) in CD8^Dual^ mice with the indicated MHC deficiencies. CD8β histograms are shown in Extended Data Fig. 1d (WT: T (*n* = 6), L (*n* = 9), MHC-II^KO^: T (*n* = 9), L (*n* = 9), β2m^KO^: T (*n* = 7), L (*n* = 9), MHC^KO^: T (*n* = 3), L (*n* = 5), 3–7 independent experiments). **e**, Mean fluorescence intensity (MFI) of Runx3d-YFP and ThPOK-GFP reporter expression in CD8.1, CD8.2, CD4^+^ and CD8^+^ T cells among TCR^hi^ thymocytes and TCR^+^ LN T cells in CD8^Dual^ and B6 mice based on histograms shown in Extended Data Fig. 1e (Runx3d-YFP: CD8^Dual^ T (*n* = 4), L (*n* = 4), B6 T (*n* = 5), L (*n* = 4), ThPOK-GFP: CD8^Dual^ T (*n* = 3), L (*n* = 3), B6 T (*n* = 4) L (*n* = 3), representative of 3–5 independent experiments). **f**, RNA-seq analysis of CD62L^+^TCR^+^ LN T cells from CD8^Dual^ and B6 mice. Genes differentially expressed between B6 CD4^+^ and CD8^+^ LN T cells were evaluated in the heat map for analysis of gene expression in CD8.1 and CD8.2 LN T cells from CD8^Dual^ mice (*n* = 3 per group, *P* < 0.05, 5-fold change). Numbers within profiles and histograms indicate frequency of cells in each box or lines (**b** and **c**). ***P < 0.001, **P < 0.01, *P < 0.05 (two-tailed unpaired *t*-tests); mean ± s.e.m. (**d** and **e**).

To identify underlying mechanisms responsible for different CD8^+^ T cell lineage fates, we constructed CD8^Dual^ mice whose *Cd4* and *Cd8* gene loci both encode CD8 co-receptors. The *Cd4* gene locus was modified to encode CD8α.1 and CD8β proteins instead of CD4 proteins, while the *Cd8* gene locus was left intact^20^. In homozygous CD8^Dual^ mice, *Cd4* gene loci encoded CD8 co-receptors (*Cd4*^CD8^) composed of CD8α.1CD8β dimers (referred to as CD8.1 co-receptors) and *Cd8* gene loci encoded CD8 co-receptors (*Cd8*^CD8^) composed of CD8α.2CD8β dimers (referred to as CD8.2 co-receptors; Fig. 1a). CD8.1 and CD8.2 co-receptors differ only in a single CD8α amino acid that does not affect CD8 co-receptor function^22^.

**Extended Data Fig. 1:**
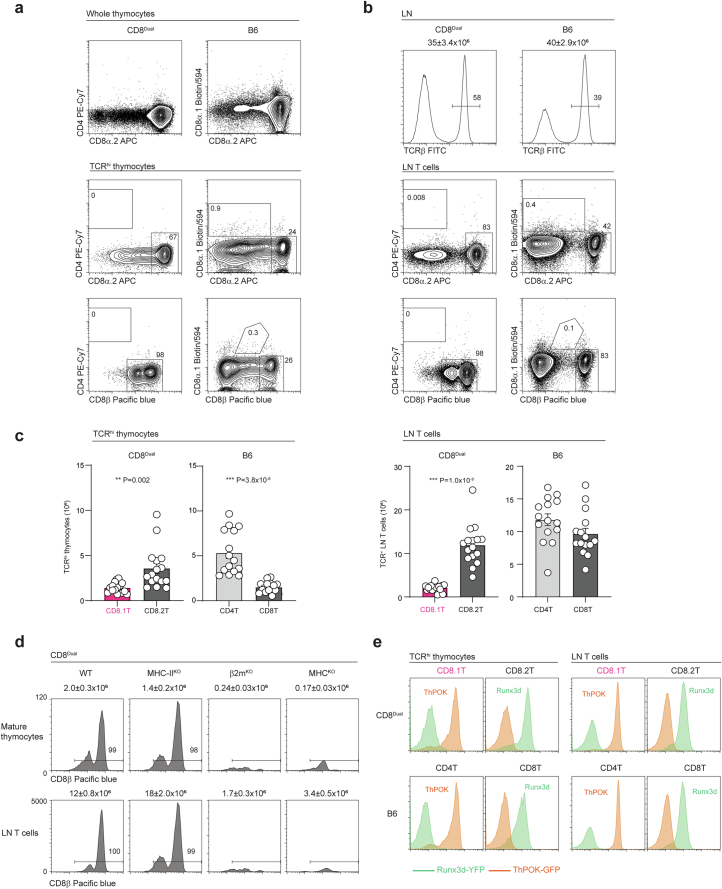
T cell development in CD8^Dual^ mice. **a**. Flow cytometry analysis of CD8^Dual^ (n = 16) and B6 (n = 15) thymocytes (representative of 15 independent experiments). **b**. Flow cytometry analysis of CD8^Dual^ (n = 16) and B6 (n = 15) LN T cells (representative of 15 independent experiments). Numbers (mean±s.e.m.) of total LN cells are shown above top histogram. **c**. Quantification of mature TCR^hi^ thymocytes and LN T cells, related to Fig. 1c. ***P < 0.001, **P < 0.01 (two-tailed unpaired *t*-tests); mean±s.e.m. **d**. CD8β expression of CD24^−^TCR^+^ mature thymocytes and TCR^+^ LN T cells from CD8^Dual^ mice with the indicated MHC deficiencies, related to Fig. 1d. **e**. Runx3d-YFP and ThPOK-GFP reporter expression among TCR^hi^ thymocytes and TCR^+^ LN T cells in CD8^Dual^ and B6 mice, related to Fig. 1e. Numbers within profiles and histograms indicate frequency of cells in each box or division (a,b,d).

Expression of CD8.1 and CD8.2 co-receptors defined four thymocyte subsets in CD8^Dual^ mice that are analogous to those defined by CD4^+^ and CD8^+^ co-receptors in wild-type (WT) C57BL/6 (B6) mice (Fig. 1b). CD8^Dual^ thymocytes consisted of CD8.1^−^8.2^−^ double-negative cells, CD8.1^+^8.2^+^ double-positive cells and CD8.1^+^8.2^−^ and CD8.1^−^8.2^+^ single-positive cells (referred to simply as CD8.1 and CD8.2 cells; Fig. 1b,c and Extended Data Fig. 1a). Notably, CD8.1 and CD8.2 cells are distinct subsets of mature TCR^hi^ thymocytes that emigrate into peripheral lymph nodes (LNs) as CD8.1 and CD8.2 T cells (Fig. 1c and Extended Data Fig. 1b,c). We found that CD8.1 and CD8.2 T cells were both selected by MHC-I-specific TCRs, as generation of all CD8^Dual^ T cells was abrogated by MHC-I deficiency (β2m^KO^) but their generation was not significantly reduced by MHC-II deficiency (MHC-II^KO^; Fig. 1d and Extended Data Fig. 1d). Thus, CD8.1 and CD8.2 T cells both express MHC-I-specific TCR and CD8 co-receptors.

### Distinct lineage fates of CD8.1 and CD8.2 T cells

**Extended Data Fig. 2:**
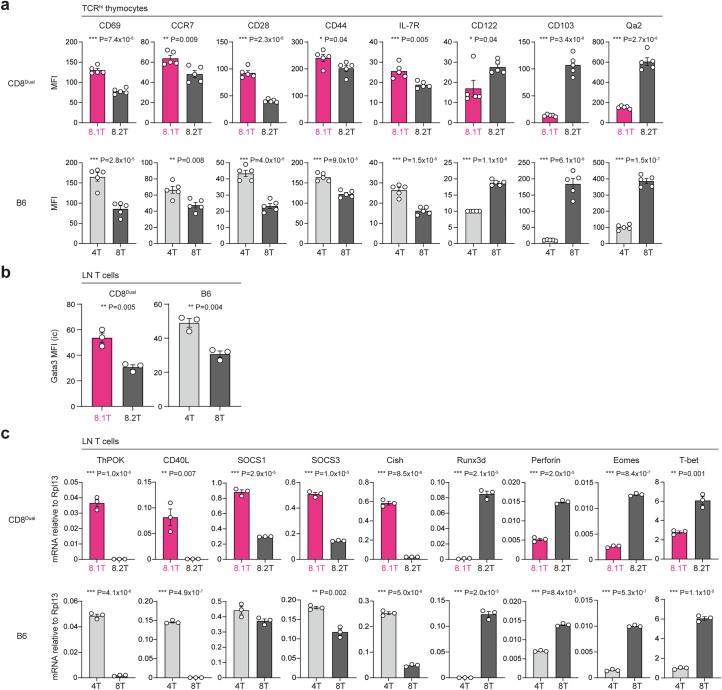
Characterization of CD8^Dual^ T cells. **a**. MFI of surface proteins on TCR^hi^ thymocytes from CD8^Dual^ and B6 mice (n = 5/strain, 5 experiments). **b**. MFI of Gata3 in LN T cells from CD8^Dual^ and B6 mice (n = 3/strain, 3 independent experiments). **c**. QPCR analysis of mRNA expression in electrically sorted LN T cells from CD8^Dual^ and B6 mice. Results are normalized to control gene *Rpl13* (n = 3/strain, 3 independent experiments with technical triplicates). ***P < 0.001, **P < 0.01, *P < 0.05 (two-tailed unpaired *t*-tests); mean±s.e.m (a-c).

Even though CD8.1 and CD8.2 T cells both express MHC-I-specific TCR and CD8 co-receptors, they expressed opposite T cell lineage factors in that *Cd4*-encoded CD8.1 T cells express helper factor ThPOK, and *Cd8*-encoded CD8.2 T cells express cytotoxic factor Runx3d (Fig. 1e and Extended Data Fig. 1e). Moreover, genome-wide RNA sequencing (RNA-seq) revealed that the transcriptional profile of CD8.1 T cells resembled that of B6 CD4^+^ helper T cells, while the transcriptional profile of CD8.2 T cells resembled that of B6 CD8^+^ cytotoxic T cells (Fig. 1f). In addition, the molecular expression pattern of CD8.1 T cells resembled that of B6 CD4^+^ helper T cells^17,23–25^, while the molecular expression pattern of CD8.2 T cells resembled that of B6 CD8^+^ cytotoxic T cells (Extended Data Fig. 2a–c)^26–30^. Thus, the different molecular expression patterns of helper lineage T cells and cytotoxic lineage T cells do not result from their expression of different co-receptor proteins but result from their expression of co-receptors encoded in different *Cd4*/*Cd8* co-receptor gene loci, as helper lineage CD8.1 T cells express co-receptors encoded in *Cd4* gene loci and cytotoxic lineage CD8.2 T cells express co-receptors encoded in *Cd8* gene loci.

**Fig. 2:**
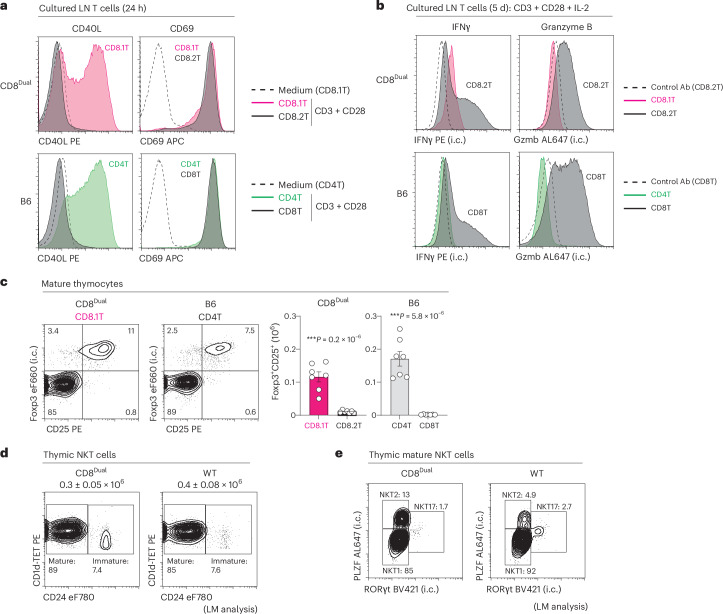
Effector functions of CD8.1 and CD8.2 CD8^Dual^ T cells. **a**, CD40L and CD69 expression on in vitro-stimulated CD8.1 and CD8.2 LN T cells from CD8^Dual^ mice, or on in vitro-stimulated CD4^+^ and CD8^+^ LN T cells from B6 mice. Magnetic activated cell sorting-purified T cells from pooled LNs were cultured with medium or plate-bound anti-CD3 + CD28 monoclonal antibodies (mAbs) for 24 h. Dashed line indicates CD8.1 or CD4^+^ T cells cultured with medium for 24 h (*n* = 4 per group, representative of 4 independent experiments. **b**, IFNγ and granzyme B (Gzmb) expression on in vitro-stimulated CD8.1 and CD8.2 LN T cells from CD8^Dual^ mice, or on in vitro-stimulated CD4^+^ and CD8^+^ LN T cells from B6 mice. Electrically sorted naive T cells (CD62L^+^CD44^−^) from pooled LNs were cultured with plate-bound anti-CD3 + CD28 mAbs for 3 days and further cultured with IL-2 for 2 days. On day 5, cells were stimulated with PMA + ionomycin for 4 h with GolgiStop. Dashed line indicates staining of CD8.2 or CD8^+^ T cells with control Abs (*n* = 3 per group, representative of 3 independent experiments). **c**, Foxp3 (intracellular or i.c.) and CD25 staining and numbers of Foxp3^+^CD25^+^ T_reg_ cells among CD24^−^TCR^+^ mature thymocytes from CD8^Dual^ and B6 mice (*n* = 7 per strain, 7 independent experiments). Foxp3 and CD25 staining among CD8^Dual^ CD8.2 and B6 CD8^+^ mature thymocytes is shown in Extended Data Fig. 4a. **d**, CD1d tetramer (CD1d-TET) and CD24 staining among CD1d-TET^+^TCR^+^ thymic NKT cells (Extended Data Fig. 4b) from CD8^Dual^ (*n* = 10) and littermate (LM) control WT (*n* = 8) mice (representative of 6 independent experiments). Numbers of thymic NKT cells (mean ± s.e.m.) are shown above profiles. **e**, Staining (i.c.) of PLZF and RORγt among CD1d-TET^+^CD24^−^ mature thymic NKT cells from CD8^Dual^ (*n* = 10) and LM control WT (*n* = 8) mice (representative of 6 independent experiments). Numbers within profiles and histograms indicate frequency of cells in each box or lines (**c**–**e**). ***P < 0.001, **P < 0.01, *P < 0.05 (two-tailed unpaired *t*-tests); mean ± s.e.m. (**c**).

**Extended Data Fig. 3:**
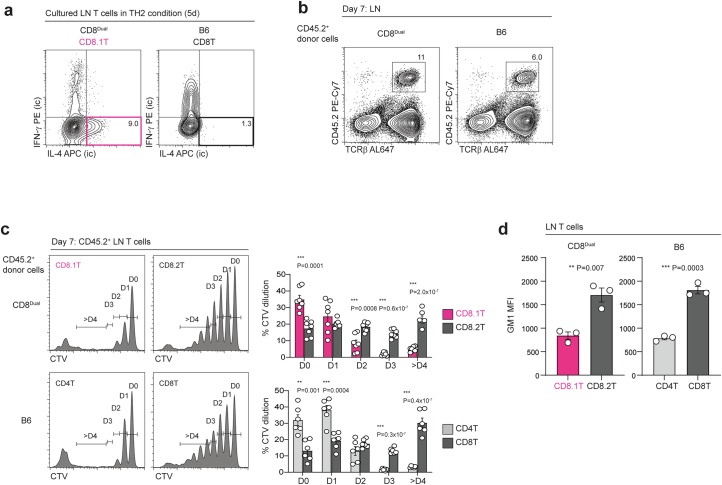
Functional assessment of CD8^Dual^ T cells. **a**. Intracellular staining (ic) of IFN-γ and IL-4 on CD8^Dual^ CD8.1 T cells and B6 CD8 T cells in Th2 culture conditions. Electrically sorted naïve T cells (CD62L^+^CD44^−^) from pooled LNs were cultured in Th2 conditions for 5 days. At day5, cells were stimulated with PMA+Ionomycin for 4 h with Golgi stop (n = 3/strain, representative of 3 independents). **b**. CD45.2 donor LN T cells from CD8^Dual^ (n = 7) and B6 (n = 6) mice were injected into sublethally irradiated CD45.1 B6 WT host mice and their LNs were characterized on day 7 (representative of 3 independent experiments). **c**. Lymphopenia-induced homeostatic proliferation of CD8^Dual^ (n = 7) and B6 (n = 6) LN T cells. CD45.2 donor LN T cells that were injected into sublethally irradiated CD45.1 B6 WT host mice were assayed at day 7 for proliferation based on CTV dilution profile (representative of 3 independent experiments). Right, frequency of CTV dilution was analyzed (right, D0: no division, D1: one division, D2: two divisions, D3: three divisions, and >D4: more than four divisions). **d**. MFI of GM1 in LN T cells from CD8^Dual^ and B6 mice (n = 3/strain, 3 independent experiments). Numbers within profiles indicate frequency of cells in each box (a,b). ***P < 0.001, **P < 0.01, *P < 0.05 (two-tailed unpaired *t*-tests); mean±s.e.m (c,d).

We assessed the cellular function of CD8.1 and CD8.2 T cells by in vitro antibody stimulation and found that anti-CD3/CD28 stimulation induced only CD8.1 T cells to express the helper T cell molecule CD40L and that type 2 helper T (T_H_2) cell stimulation cultures induced CD8.1 T cells to become IL-4^+^ T_H_2 cells (Fig. 2a and Extended Data Fig. 3a). In contrast, anti-CD3/CD28 stimulation in IL-2 cultures induced only CD8.2 T cells to express the cytotoxic T cell molecules IFNγ and granzyme B (Fig. 2b). Moreover, injection of CD45.2 CD8^Dual^ T cells into sublethally irradiated lymphopenic CD45.1 host mice caused CD8.1 T cells to undergo limited lymphopenic proliferation like that of B6 CD4^+^ helper T cells, but caused CD8.2 T cells to undergo extensive lymphopenic proliferation like that of B6 CD8^+^ cytotoxic T cells, which correlated with their significantly higher GM1 staining of lipid raft components (Extended Data Fig. 3b–d)^31,32^. Thus, CD8.1 T cells that express *Cd4*-encoded co-receptors possess helper function and CD8.2 T cells that express *Cd8*-encoded CD8 co-receptors possess cytotoxic function.

**Extended Data Fig. 4:**
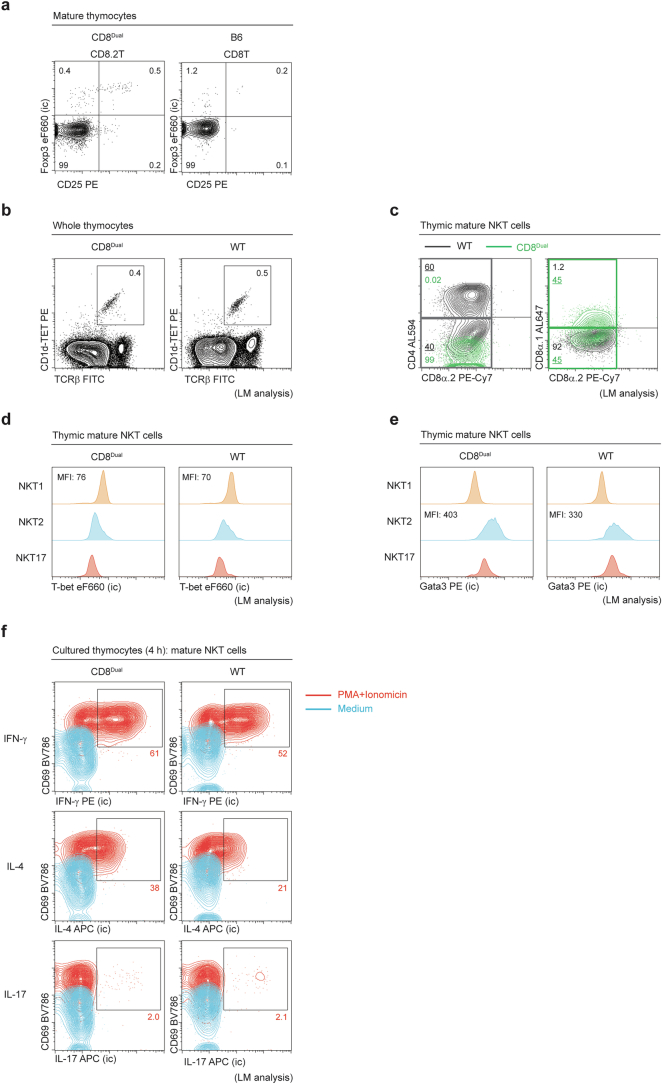
Agonist-selected T cells in CD8^Dual^ mice. **a**. Foxp3 (ic) and CD25 staining among mature CD8.2 and CD8 thymocytes from CD8^Dual^ and B6 mice, related to Fig. 2c. **b**. CD1d-TET and TCRβ staining of whole thymocytes from CD8^Dual^ and LM control WT mice, related to Fig. 2d. **c**. Flow cytometry analysis of CD1d-TET^+^CD24^−^ thymic mature NKT cells from CD8^Dual^ (green) and LM control WT (gray) mice (n = 3/strain, representative of 3 independent experiments). **d**. Intracellular staining (ic) of T-bet in NKT subsets from CD8^Dual^ and LM control WT mice (n = 3/strain, representative of 3 independent experiments). **e**. Intracellular staining (ic) of Gata3 in NKT subsets from CD8^Dual^ and LM control WT mice (n = 3/strain, representative of 3 independent experiments). **f**. IFN-γ, IL-4, and IL-17 expression in CD1d-TET^+^CD24^−^ thymic mature NKT cells from CD8^Dual^ and LM control WT mice. Whole thymocytes were cultured with medium or PMA+Ionomycin in the presence of golgi stop for 4 h and assessed for cytokine production (n = 3/strain, representative of 3 independent experiments). Numbers within profiles indicate frequency of cells in each box (a-c,f).

Finally, because CD4^+^ helper lineage T cells differentiate into Foxp3^+^ regulatory T (T_reg_) cells^33,34^ and PLZF^+^ invariant natural killer T (NKT) cells in B6 thymi^35–38^, we asked if CD8.1 helper lineage T cells also differentiate into T_reg_ cells and NKT cells in CD8^Dual^ thymi. Indeed, CD8^Dual^ thymi contain Foxp3^+^CD8.1 T_reg_ cells (Fig. 2c and Extended Data Fig. 4a) and contain all three NKT subsets (NKT1, NKT2 and NKT17) of CD8.1 NKT cells that resemble those in WT thymi in that NKT1 cells are T-bet^+^IFNγ^+^; NKT2 cells are Gata3^+^IL-4^+^; and NKT17 cells are RORγt^+^IL-17^+^ (Fig. 2d,e and Extended Data Fig. 4b–f)^39,40^. Notably, CD8^Dual^ thymi contained 2–3-fold higher frequencies of NKT2 cells than WT B6 thymi (Fig. 2e).

Thus, despite expressing MHC-I-specific TCR and CD8 co-receptors, CD8^Dual^ thymocytes differentiate into two distinct mature T cell subsets: CD8.1 T cells, which are ThPOK^+^ helper lineage cells, and CD8.2 T cells, which are Runx3d^+^ cytotoxic lineage cells. These two CD8^+^ T cell subsets display different gene profiles, different molecular expression patterns and different cellular functions. Thus, even when they encode the same CD8 co-receptor, *Cd4* gene loci promote generation of helper CD8^+^ T cells, and *Cd8* gene loci promote generation of cytotoxic CD8^+^ T cells.

### Co-receptor kinetics and signaling duration during positive selection

**Fig. 3:**
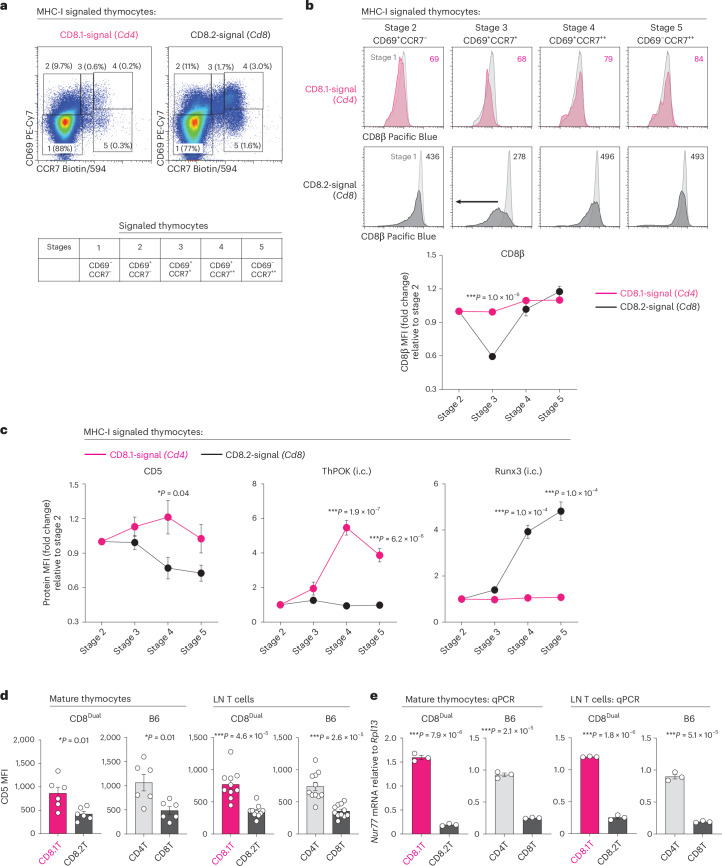
Positive selection of MHC-I signaled CD8.1 and CD8.2 thymocytes. **a**, CD69 versus CCR7 profiles identify thymocytes at sequential stages of positive selection, with TCR-unsignaled cells in stage 1 and TCR-signaled cells undergoing positive selection in stages 2–5. Numbers in parentheses indicate frequency of cells at each sequential stage. Left: MHC-I/CD8.1 signaled thymocytes were assessed in CD8^Dual^ CD8α.2^KO^MHC-II^KO^CD1d^KO^ thymocytes (*n* = 6), which express only *Cd4*-encoded CD8.1 co-receptors. Right: MHC-I/CD8.2 signaled thymocytes were assessed in MHC-II^KO^CD1d^KO^ thymocytes (*n* = 7), which express *Cd8*-encoded CD8.2 co-receptors (representative of 6 independent experiments). **b**, Kinetics of surface CD8β co-receptor expression at different stages of positive selection of MHC-I/CD8.1 signaled thymocytes (top row, *n* = 6) and MHC-I/CD8.2 signaled thymocytes (2nd row, *n* = 6) as in **a**. CD8β surface expression at each stage is overlaid on stage 1 thymocytes, and the numbers in each panel indicate CD8β MFI. Bottom: CD8β MFI values of thymocytes at each stage are normalized to those of stage 2 thymocytes, which are set equal to 1.0 (representative of 4 independent experiments). **c**, Kinetics of CD5, ThPOK and Runx3 expression during MHC-I/CD8.1 signaled positive selection and MHC-I/CD8.2 signaled positive selection. MFI of each protein was normalized to stage 2 thymocytes, which were set equal to 1.0 (MHC-I/CD8.1 signaled thymocytes: CD5 (*n* = 6), ThPOK (*n* = 6), Runx3 (*n* = 6), MHC-I/CD8.2 signaled thymocytes: CD5 (*n* = 5), ThPOK (*n* = 7), Runx3 (*n* = 7), 4–6 independent experiments). **d**, CD5 MFI on CD24^−^TCR^+^ mature thymocytes and TCR^+^ LN T cells from the indicated mice (CD8^Dual^: T (*n* = 6), L (*n* = 10), B6: T (*n* = 6), L (*n* = 11), 6–11 independent experiments). **e**, *Nur77* mRNA expression in electrically sorted CD24^−^TCR^+^ mature thymocytes and TCR^+^ LN T cells from CD8^Dual^ and B6 mice. Results are relative to control *Rpl13* gene (*n* = 3 per group, 3 independent experiments with technical triplicates). ***P < 0.001, **P < 0.01, *P < 0.05 (two-tailed unpaired *t*-tests); mean ± s.e.m. (**b**–**e**).

In WT mice, *Cd4* and *Cd8* gene loci regulate MHC-I and MHC-II TCR signaling duration during positive selection by differentially regulating the kinetics of CD4 and CD8 co-receptor expression^13^. Consequently, we assessed if *Cd4* and *Cd8* gene loci regulate MHC-I TCR signaling duration during positive selection in CD8^Dual^ mice by differentially regulating the kinetics of expression of CD8.1 and CD8.2 co-receptors, respectively. To do so, we examined MHC-I signaled thymocytes expressing CD8.1 or CD8.2 co-receptors at five sequential stages of positive selection as defined by CD69 and CCR7 surface expression^33,41^: CD69^−^CCR7^−^ stage 1 cells are TCR-unsignaled thymocytes; CD69^+^CCR7^−^ stage 2 cells are thymocytes that have just been TCR-signaled; and CD69^+^CCR7^+^ and CD69^−^CCR7^+^ cells are thymocytes at subsequent stages 3–5 of positive selection (Fig. 3a). We found that CD8.2 surface expression acutely declined on stage 3 thymocytes, whereas CD8.1 surface expression steadily increased (Fig. 3b), which are concordant with the kinetic signaling concept that *Cd8* gene expression is specifically but transiently terminated in TCR-signaled thymocytes^13^. To determine how these different co-receptor kinetics affected TCR signaling, we normalized CD5 expression to TCR-signaled stage 2 thymocytes (Fig. 3c)^42^. Notably, CD5 expression between stages 2 and 4 increased on CD8.1 thymocytes but decreased on CD8.2 thymocytes (Fig. 3c), verifying that MHC-I TCR signaling that is dependent on *Cd4*-encoded CD8.1 co-receptors persisted without disruption, whereas TCR signaling that is dependent on *Cd8*-encoded CD8.2 co-receptors underwent disruption. Importantly, persistence of *Cd4*-dependent MHC-I TCR signaling induced ThPOK (Fig. 3c), while disruption of *Cd8*-dependent TCR signaling resulted in Runx3 expression (Fig. 3c). Note that CD5 expression always declined at stage 5 due to TCR signaling termination, which is necessary for thymocytes to express the chemotactic receptor S1P1 and exit the thymus (Fig. 3c)^43–45^. Nevertheless, CD5 expression remained higher on CD8.1 than CD8.2 mature thymocytes even after they exited the thymus and became CD8.1 and CD8.2 LN T cells (Fig. 3d). Interestingly, we found that *Nur77* mRNA was also more highly expressed in CD8.1 than CD8.2 mature thymocytes, and this difference also persisted in LN T cells (Fig. 3e)^46^. Thus, CD5 and Nur77 expression on mature thymocytes and peripheral T cells reflect TCR signaling duration during positive selection.

**Extended Data Fig. 5:**
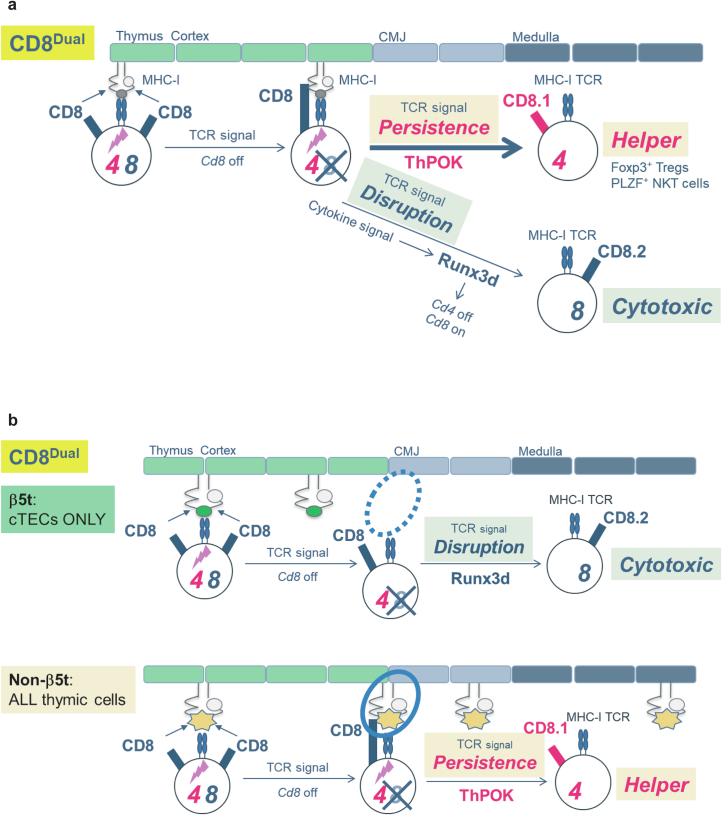
Schematic of CD8 T-lineage fate decisions in the CD8^Dual^ thymus. **a**. CD8 T-lineage fate decisions in the CD8^Dual^ thymus. TCR signaling transcriptionally terminates *Cd8* gene expression (not *Cd4* gene expression) which reduces surface expression of *Cd8*-encoded CD8 coreceptors but upregulates expression of *Cd4*-encoded CD8 coreceptors. Consequently, MHC-I TCR signaling that becomes disrupted allows thymic cytokines to induce the cytotoxic factor Runx3d that silences *Cd4* gene expression, re-initiates *Cd8* gene expression, and promotes thymocyte differentiation into cytotoxic T cells. In contrast, MHC-I TCR signaling that persists undisrupted induces the helper factor ThPOK that promotes thymocyte differentiation into helper T cells. **b**. MHC-I peptides affect CD8-T lineage fate decisions in the CD8^Dual^ thymus. cTECs in the thymic cortex express two classes of peptides which we refer to as β5t- and nonβ5t-peptides which display different patterns of expression. β5t-peptides are exclusively expressed on cTECs in the thymic cortex, whereas nonβ5t-peptides are expressed throughout the thymus. Consequently, TCR engagement of β5t-peptides on cTECs in the thymic cortex becomes permanently disrupted when thymocytes disengage from cTECs and migrate through the thymus, which induces Runx3d expression and results in generation only of cytotoxic CD8 T cells. In contrast, TCR engagement of nonβ5t-peptides on thymic cells in the cortex or CMJ can persist when thymocytes disengage from cTECs and migrate through the thymus, which induces ThPOK expression and results in generation of helper CD8 T cells.

Our findings reveal that *Cd4*-encoded CD8 co-receptors promote TCR signaling persistence, which induces ThPOK expression and helper fate; whereas *Cd8*-encoded CD8 co-receptors promote TCR signaling disruption, which results in Runx3d expression and cytotoxic fate (schematized in Extended Data Fig. 5a).

### Monoclonal TCR can induce different CD8^+^ T cell lineage fates

**Fig. 4:**
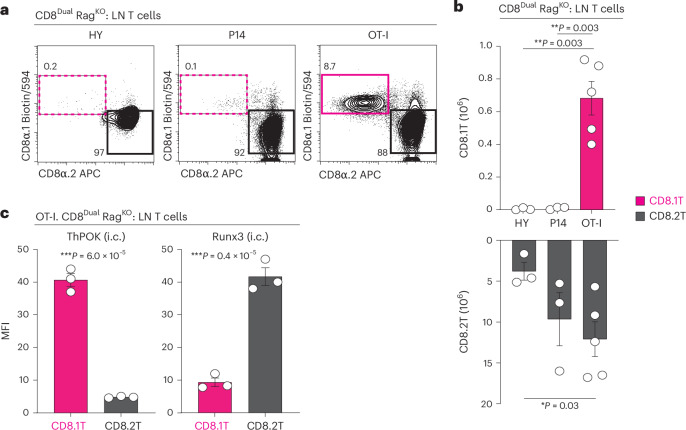
Impact of MHC-I monoclonal TCRs on T cell lineage fates in CD8^Dual^ mice. **a**, Flow cytometry showing CD8α.1 and CD8α.2 expression of T3.70^+^ (HY TCR) or Vα2^+^ (P14 and OT-I TCRs) LN T cells from CD8^Dual^ Rag^KO^ mice with monoclonal MHC-I TCR transgenes. Numbers within profiles indicate frequency of cells in each box (HY (*n* = 3), P14 (*n* = 3), OT-I (*n* = 5), 3–5 independent experiments). **b**, Numbers of CD8.1 and CD8.2 LN T cells from CD8^Dual^ Rag^KO^ mice with monoclonal MHC-I TCR transgenes (HY (*n* = 3), P14 (*n* = 3), OT-I (*n* = 5), 3–5 independent experiments). **c**, MFI of ThPOK and Runx3 in CD8.1 and CD8.2 T cells among Vα2^+^ LN T cells from OT-I. CD8^Dual^ Rag^KO^ mice (*n* = 3, 3 independent experiments). ***P < 0.001, **P < 0.01, *P < 0.05 (two-tailed unpaired *t*-tests); mean ± s.e.m. (**b** and **c**).

Having documented that CD8^Dual^ thymocytes are signaled during positive selection to adopt helper or cytotoxic lineage fates depending on whether MHC-I TCR signaling persists or undergoes disruption, we wanted to determine how monoclonal MHC-I-specific TCR would affect CD8^+^ T cell lineage fate decisions in CD8^Dual^ mice. To answer this question, we bred three different MHC-I-specific TCR transgenes (OT-I, P14 and HY) into CD8^Dual^ Rag^KO^ mice (Fig. 4a)^47–49^. Expression of low-affinity HY and P14 TCR generated only CD8.2 cytotoxic T cells, which indicated that their signaling had been disrupted during positive selection (Fig. 4a,b). In contrast, expression of high-affinity OT-I TCR generated both CD8.1 T cells that expressed ThPOK and CD8.2 T cells that expressed Runx3 (Fig. 4a–c). That OT-I TCR generated CD8.1 helper T cells, but HY and P14 TCR did not, indicated that signaling persistence required higher-affinity TCR than signaling disruption, which was not surprising. However, the fact that monoclonal OT-I TCR generated both helper and cytotoxic CD8^+^ T cells indicated that OT-I thymocytes experienced different duration TCR signaling despite identical OT-I TCR and CD8 co-receptors, suggesting that another as-yet-unknown factor influenced MHC-I TCR signaling duration in the thymus.

### Contribution of thymic selecting peptides to different CD8^+^ T cell lineage fates

Because MHC-I TCRs engage thymic MHC-I–peptide complexes to signal positive selection^50,51^, we wondered if TCR engagement of different thymic MHC-I peptides might generate different duration MHC-I TCR signaling. Because TCR engagement of MHC-I–peptide complexes on cortical thymic epithelial cells (cTECs) signals thymocytes to undergo positive selection and to migrate to the corticomedullary junction and medulla^52–56^, we thought that TCR signaling duration might depend on whether TCR–ligand engagements that are initiated in the cortex persist or become disrupted after thymocytes disengage from the cortex and migrate to the corticomedullary junction and medulla.

Notably, thymic MHC-I peptides are produced in cTECs by proteasomes with different β5 proteasomal subunits, in that ‘thymoproteasomes’ contain β5t subunits and produce β5t-peptides, whereas other proteasomes produce nonβ5t-peptides^57,58^. Because thymoproteasomes are expressed exclusively in cTECs, the β5t-peptides they produce are only present on cTECs; and because other proteasomes are expressed in both cTECs and other thymic cellular elements, the nonβ5t-peptides they produce are expressed on both cTECs and other thymic cellular elements. Consequently, TCR engagement of β5t-peptide–MHC-I complexes must invariably be disrupted when thymocytes disengage from the thymic cortex and migrate, whereas TCR engagement of nonβ5t-peptide–MHC-I complexes might persist even after thymocytes disengage and migrate out of the thymic cortex (schematized in Extended Data Fig. 5b). Because disrupted TCR signaling during positive selection induces cytotoxic fate and undisrupted TCR signaling induces helper fate, we thought it possible that TCR engagement of β5t-peptides and nonβ5t-peptides on cTECs might induce different CD8^+^ T cell lineage fates (schematized in Extended Data Fig. 5b).

### β5t-peptides exclusively select CD8^+^ cytotoxic T cells

**Fig. 5:**
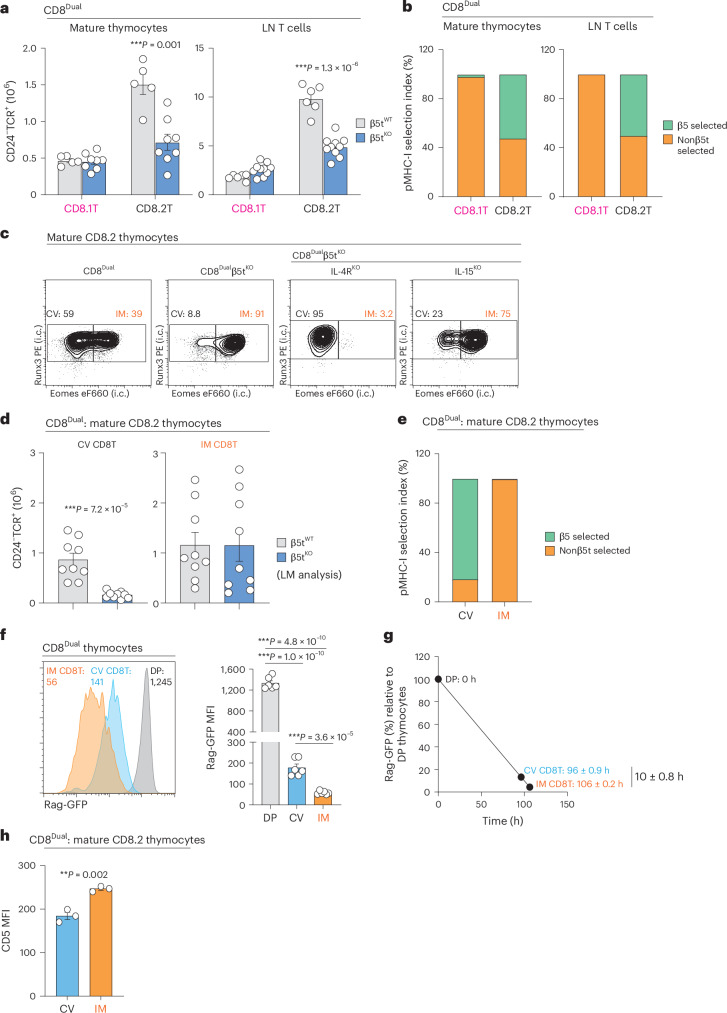
Impact of different thymic MHC-I peptides on CD8^+^ T cell lineage fate. **a**, Numbers of CD8.1 and CD8.2 T cells among CD24^−^TCR^+^ mature thymocytes and TCR^+^ LN T cells from β5t^WT^ and β5t^KO^ CD8^Dual^ mice (β5t^WT^: T (*n* = 5), L (*n* = 6), β5t^KO^: T (*n* = 8), L (*n* = 10), 5–6 independent experiments). CD8α.1 versus CD8β profiles are shown in Extended Data Fig. 6a. **b**, MHC-I peptide selection index showing the frequencies of β5t- or nonβ5t-selected cells among CD8.1 and CD8.2 T cells of CD24^−^TCR^+^ mature thymocytes and TCR^+^ LN T cells from CD8^Dual^ mice. **c**, Staining (i.c.) of Runx3 and Eomes in CD8.2 T cells among CD24^−^TCR^+^ mature thymocytes from indicated mice. Numbers within profiles indicate frequency of cells in each box (CD8^Dual^β5t^WT^: *n* = 9, CD8^Dual^β5t^KO^: *n* = 9, CD8^Dual^β5t^KO^IL-4R^KO^: *n* = 7, CD8^Dual^β5t^KO^IL-15^KO^: *n* = 6, representative of 6–7 independent experiments). **d**, Numbers of CV (Runx3^+^Eomes^−^) and IM (Runx3^+^Eomes^+^) CD8.2 T cells among CD24^−^TCR^+^ mature thymocytes from LM control β5t^WT^ and β5t^KO^ CD8^Dual^ mice (*n* = 9 per strain, 6 independent experiments). **e**, MHC-I peptide selection index showing the frequencies of β5t- and nonβ5t-selected cells in CV and IM CD8.2 T cells among CD24^−^TCR^+^ mature thymocytes from CD8^Dual^ mice. **f**, Rag-GFP reporter expression in double-positive (CD24^+^CD8.1^+^CD8.2^+^), CV CD8.2 (CXCR3^−^) and IM CD8.2 (CXCR3^+^CD44^+^) thymocytes from CD8^Dual^ mice. Numbers in histogram indicate MFI of Rag-GFP expression (*n* = 6, 3 independent experiments). **g**, Differentiation time of CV and IM CD8^+^ T cells from double-positive thymocytes was calculated based on Rag-GFP MFI (F) half-life of 54–56 h as described in the Methods. **h**, MFI of CD5 on CV (Runx3^+^Eomes^−^) and IM (Runx3^+^Eomes^+^) CD8.2 T cells among CD24^−^TCR^+^ mature thymocytes from CD8^Dual^ mice (*n* = 3 per strain, 3 independent experiments). ***P < 0.001, **P < 0.01, *P < 0.05 (two-tailed unpaired *t*-tests); mean ± s.e.m. (**a**, **d**, **f** and **h**).

**Extended Data Fig. 6:**
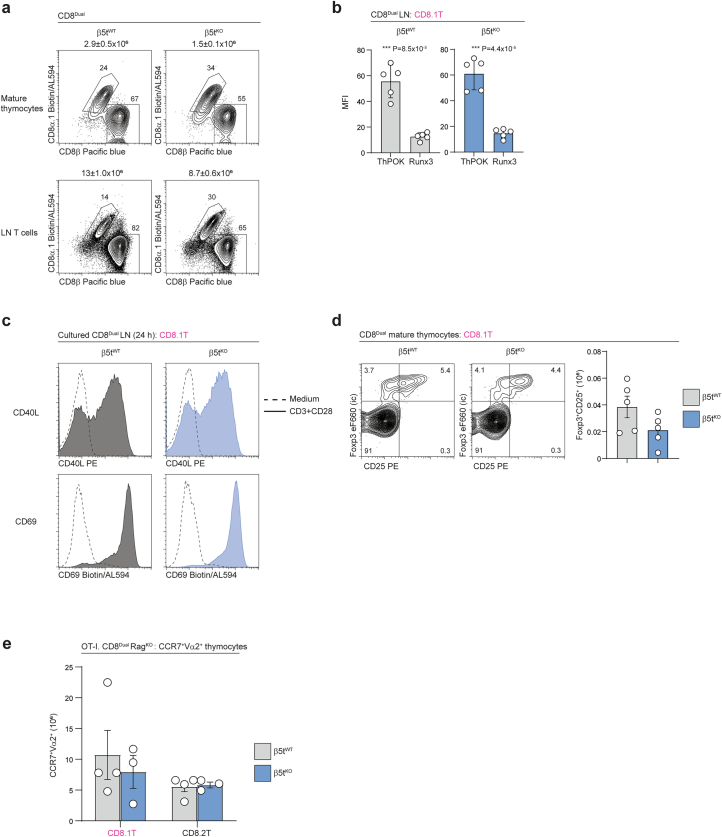
Selection of cytotoxic CD8 T cells by β5t-peptides. **a**. Flow cytometry of CD24^−^TCR^+^ mature thymocytes and TCR^+^ LN T cells from β5t^WT^ and β5t^KO^ CD8^Dual^ mice, related to Fig. 5a, b. Numbers (mean±s.e.m.) of mature thymocytes and LN T cells are shown above profiles. **b**. MFI of ThPOK and Runx3 in CD8.1 LN T cells from β5t^WT^ and β5t^KO^ CD8^Dual^ mice (n = 5/strain, 4 independent experiments). **c**. CD40L and CD69 expression on *in vitro* stimulated CD8.1 LN T cells from β5t^WT^ and β5t^KO^ CD8^Dual^ mice. MACS-purified T cells from pooled LNs were cultured with plate-bound anti-CD3 + CD28 mAbs for 24 h. Dot line indicates CD8.1 T cells cultured with medium for 24 h (n = 3/group, representative of 3 independent experiments). **d**. Foxp3 (ic) and CD25 staining and numbers of Foxp3^+^CD25^+^ mature Tregs among CD24^−^TCR^+^ mature CD8.1 thymocytes from β5t^WT^ and β5t^KO^ CD8^Dual^ mice (n = 5/strain, 4 independent experiments). **e**. Numbers of OT-I CD8.1 and OT-I CD8.2 T cells among Vα2^+^CCR7^+^ mature thymocytes from β5t^WT^ (n = 4) and β5t^KO^ OT-I. CD8^Dual^ Rag^KO^ mice (n = 3, 3 independent experiments). Numbers within profiles indicate frequency of cells in each box (a,d). ***P < 0.001, **P < 0.01, *P < 0.05 (two-tailed unpaired *t*-tests); mean±s.e.m (b,d,e).

To experimentally determine if β5t-peptides and nonβ5t-peptides induce different CD8^+^ T cell lineage fates, we compared CD8.1 helper and CD8.2 cytotoxic T cell generation in intact (β5t^WT^) and β5t-deficient (β5t^KO^) CD8^Dual^ mice (Fig. 5a). Quite unexpectedly, CD8.1 helper T cell generation was identical in β5t^WT^ and β5t^KO^ mice, which indicated that CD8.1 helper T cell generation was unaffected by the absence of β5t-peptides (Fig. 5a and Extended Data Fig. 6a). Absent β5t-peptides in β5t^KO^ mice also did not alter helper lineage features of CD8.1 T cells such as ThPOK expression, CD40L induction and Foxp3 expression (Extended Data Fig. 6b–d). In contrast, absent β5t-peptides reduced generation of CD8.2 cytotoxic T cells by ~50% in β5t^KO^ mice compared to β5t^WT^ mice, which indicated that CD8.2 cytotoxic T cells were selected by both β5t-peptides and nonβ5t-peptides (Fig. 5a). To more clearly appreciate the impact of β5t-peptides and nonβ5t-peptides on CD8^+^ T cells, we defined ‘MHC-I peptide selection index’ as the normalized percentage of T cells selected by β5t-peptides and nonβ5t-peptides, which illustrated that CD8.1 helper T cells were selected by nonβ5t-peptides, whereas CD8.2 cytotoxic T cells were selected by both β5t-peptides and nonβ5t-peptides (Fig. 5b).

We then wished to determine the thymic peptides that selected monoclonal OT-I TCR thymocytes to become CD8.1 helper or CD8.2 cytotoxic T cells (Fig. 4). We found that both OT-I CD8.1 helper and OT-I CD8.2 cytotoxic mature thymocytes were selected by nonβ5t-peptides as neither was affected by the absence of β5t-peptides in β5t^KO^ mice (Extended Data Fig. 6e). It might also be noted that more OT-I CD8.1 helpers than OT-I CD8.2 killers appeared in the thymus, whereas the reverse was the case among peripheral LN T cells (Fig. 4 and Extended Data Fig. 6e), possibly because of the far greater proliferative potential in the lymphopenic periphery of CD8.2 cytotoxic than CD8.1 helper T cells (Extended Data Fig. 3c).

We conclude that different thymic MHC-I peptides induce different CD8^+^ T cell lineage fates, with β5t-peptides generating only CD8 cytotoxic T cells and nonβ5t-peptides generating either helper or cytotoxic CD8^+^ T cells.

### IM CD8^+^ T cells are only selected by nonβ5t-peptides

**Extended Data Fig. 7:**
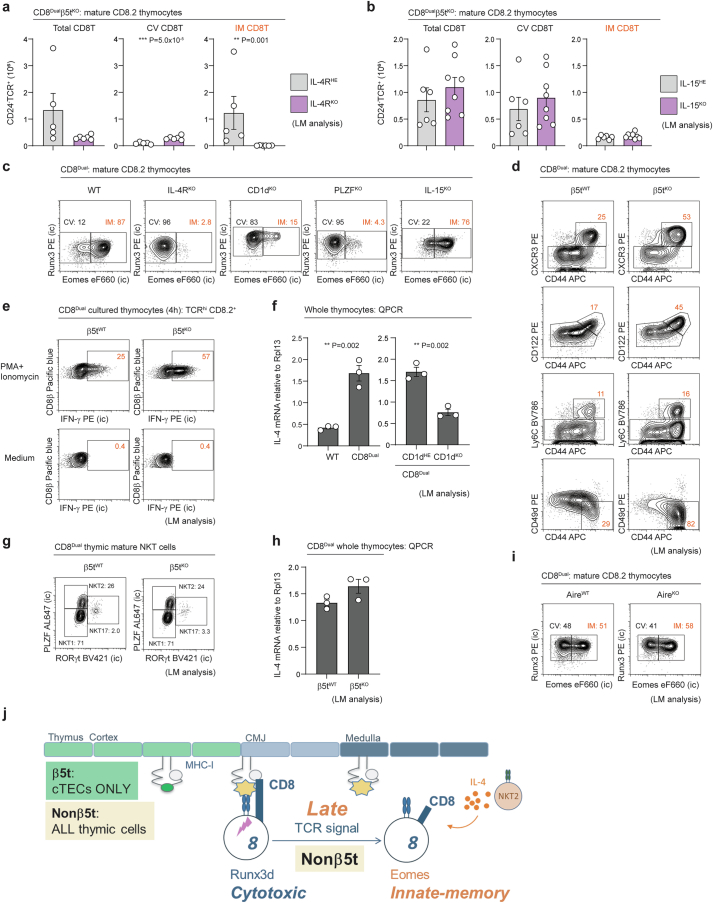
Selection of IM-CD8 T cells by nonβ5t-peptides. **a**. Numbers of total, CV ((Runx3^+^Eomes^−^), and IM (Runx3^+^Eomes^+^) CD8.2 T cells among mature thymocytes from LM control IL-4R heterozygous (IL-4R^HE^: n = 5) and IL-4R^KO^ (n = 6) CD8^Dual^β5t^KO^ mice, related to Fig. 5c, third profile (5-6 independent experiments). **b**. Numbers of total, CV, and IM CD8.2 T cells among mature thymocytes from LM control IL-15^HE^ (n = 6) and IL-15^KO^ (n = 8) CD8^Dual^ β5t^KO^ mice, related to Fig. 5c, fourth profile (5 independent experiments). **c**. Runx3 and Eomes expression on mature CD8.2 thymocytes from indicated CD8^Dual^ mice (IL-4R^KO^: n = 7, CD1d^KO^: n = 3, PLZF^KO^: n = 5, IL-15^KO^: n = 3, 3-4 independent experiments). **d**. Flow cytometry of mature CD8.2 thymocytes from LM control β5t^WT^ and β5t^KO^ CD8^Dual^ mice (n = 3/strain, 3 independent experiments). **e**. IFN-γ expression by TCR^hi^ CD8.2 thymocytes from LM control β5t^WT^ and β5t^KO^ CD8^Dual^ mice. Whole thymocytes were cultured with medium or PMA+Ionomycin for 4 h in the presence of golgi stop (n = 3/strain, 3 independent experiments). **f**. QPCR analysis of IL-4 mRNA in whole thymocytes from indicated mice. Results are normalized to control gene *Rpl13* (n = 3/strain, 3 independent experiments with technical triplicates). **g**. PLZF and RORγt expression of thymic mature NKT cells from LM control β5t^WT^ (n = 4) and β5t^KO^ (n = 5) CD8^Dual^ mice (3 independent experiments). **h**. QPCR analysis of IL-4 mRNA in whole thymocytes from LM control β5t^WT^ and β5t^KO^ CD8^Dual^ mice. Results are normalized to control gene *Rpl13* (n = 3/strain, 3 independent experiments with technical triplicates). **i**. Runx3 and Eomes expression on mature CD8.2 thymocytes from LM control Aire^WT^ and Aire^KO^ CD8^Dual^ mice (n = 8/strain, 4-5 independent experiments). **j**. IM-CD8 T cell generation requires late-TCR signaling by nonβ5t-peptides. If TCR engagement of nonβ5t-peptides on cTECs is transiently disrupted by reduced CD8 coreceptor expression, thymocytes express Runx3d and become cytotoxic CD8 T cells. Because Runx3d re-initiates *Cd8* gene expression and upregulates surface CD8 coreceptor expression, TCR can subsequently re-engage nonβ5t-peptides when they encounter them on non-cortical thymic elements, stimulating late-TCR signaling. Late-TCR signaling may delay thymocytes from leaving the thymus which prolongs their exposure to IL-4 produced by NKT2 cells in the thymic medulla. Numbers within profiles indicate frequency (c-e,g,i). ***P < 0.001, **P < 0.01 (two-tailed unpaired *t*-tests); mean±s.e.m (a,b,f,h).

To focus specifically on thymic selection of CD8 cytotoxic T cells, we compared CD8.2 cytotoxic thymocytes from β5t^WT^ and β5t^KO^ CD8^Dual^ mice (Fig. 5c–e). Most (~60%) CD8.2 thymocytes in β5t^WT^CD8^Dual^ mice were conventional (CV) CD8^+^ T cells in that they were Runx3^+^Eomes^−^, whereas nearly all (>90%) CD8.2 thymocytes in β5t^KO^ CD8^Dual^ mice were IM CD8^+^ T cells that were Runx3^+^Eomes^+^ (Fig. 5c–e)^39,59,60^. Generation of nonβ5t-selected IM CD8^+^ T cells in CD8^Dual^ mice resembled generation of IM CD8^+^ T cells in WT mice in being IL-4-dependent and abrogated in IL-4R^KO^ but not IL-15^KO^ mice, and in requiring NKT cells, which are absent in PLZF^KO^ and CD1d^KO^ CD8^Dual^ mice (Fig. 5c and Extended Data Fig. 7a–c)^39,59,60^. Moreover, nonβ5t-selected IM CD8^+^ T cells in CD8^Dual^ mice are CD44^hi^, CXCR3^+^, CD122^+^, Ly6C^+^ and CD49d^−^ and produce IFNγ after PMA + ionomycin stimulation (Extended Data Fig. 7d,e)^60,61^. Importantly, because of their 2–3-fold greater frequency of NKT2 cells, CD8^Dual^ thymi express significantly more IL-4 mRNA than WT B6 thymi (Fig. 2e and Extended Data Fig. 7f). Notably, β5t deficiency affected neither thymic NKT2 cell frequencies nor IL-4 mRNA amounts (Extended Data Fig. 7g,h). We conclude that β5t-peptides select CV CD8^+^ T cells, whereas IM CD8^+^ T cells are selected by nonβ5t-peptides.

Because nonβ5t-selected CD8^+^ cytotoxic thymocytes could reencounter their selecting peptides outside the thymic cortex, differentiation into IM CD8^+^ T cells might be induced by late TCR signaling stimulated by nonβ5t-peptides on non-cortical cells. If this were the case, β5t-selected CV CD8^+^ T cells would be predicted to arise earlier than nonβ5t-selected IM CD8^+^ T cells. To assess this possibility, we utilized Rag-GFP expressing CD8^Dual^ thymocytes to determine the order of appearance in the thymus of CV and IM CD8.2 T cells (Fig. 5f,g)^41,62^. We found that IM CD8.2 T cells appeared in the thymus ~10 h later and had significantly higher CD5 expression than CV CD8.2 T cells, suggesting that IM CD8^+^ T cells receive late TCR signaling stimulated by nonβ5t-peptides outside the cortex (Fig. 5f–h). We then wondered if expression of the nonβ5t-peptides that stimulated IM CD8^+^ T cell generation was dependent on Aire, which is specifically expressed in mTECs^63,64^. However, contradicting this possibility, we found that IM CD8^+^ T cell generation was unaffected by Aire deficiency (Extended Data Fig. 7i). Based on these results, we suggest that late TCR signaling is induced by nonβ5t-peptides encountered outside the thymic cortex and delays CD8^+^ thymocytes from exiting the thymus, which increases exposure to intrathymic IL-4 and promotes differentiation into IM CD8^+^ T cells (schematized in Extended Data Fig. 7j).

### Peptide selection of CD8^+^ T cells in WT mice

**Fig. 6:**
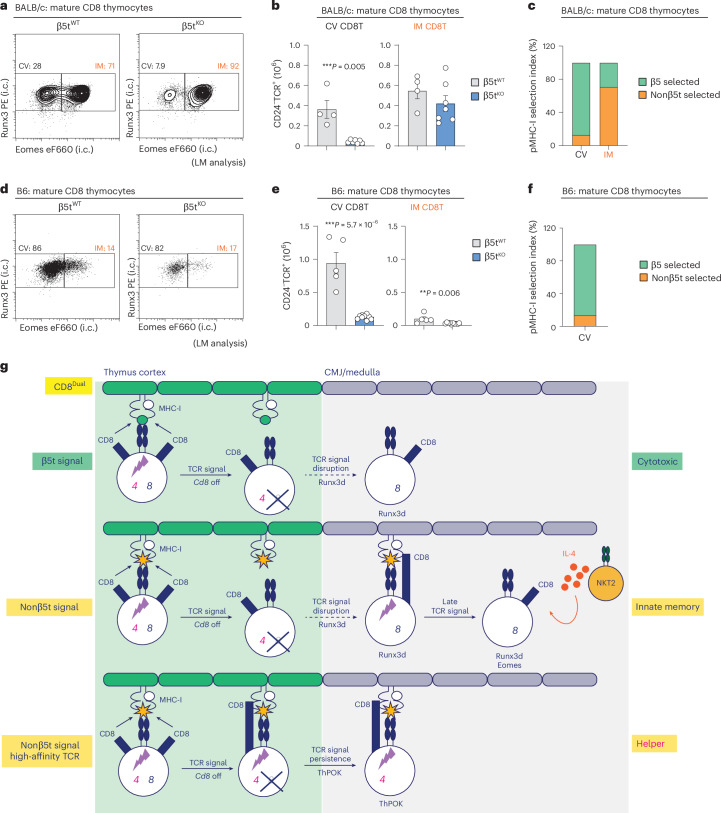
Impact of different thymic MHC-I peptides on cytotoxic CD8^+^ T cells in WT mice. **a**, Staining (i.c.) of Runx3 and Eomes in CD8^+^ T cells among CD24^−^TCR^+^ mature thymocytes from LM control β5t^WT^ (*n* = 4) and β5t^KO^ (*n* = 7) BALB/c mice (representative of 3–4 independent experiments). **b**, Numbers of CV (Runx3^+^Eomes^−^) and IM (Runx3^+^Eomes^+^) CD8^+^ T cells among CD24^−^TCR^+^ mature thymocytes from LM control β5t^WT^ and β5t^KO^ BALB/c mice (**a**). **c**, MHC-I peptide selection index showing the frequencies of β5t- and nonβ5t-selected cells among CV and IM CD8^+^ T cells in CD24^−^TCR^+^ mature BALB/c thymi. **d**, Staining (i.c.) of Runx3 and Eomes on CD8^+^ T cells among CD24^−^TCR^+^ mature thymocytes from LM control β5t^WT^ (*n* = 5) and β5t^KO^ (*n* = 10) B6 mice (representative of 5–8 independent experiments). **e**, Numbers of CV (Runx3^+^Eomes^−^) and IM (Runx3^+^Eomes^+^) CD8^+^ T cells among CD24^−^TCR^+^ mature thymocytes from LM control β5t^WT^ and β5t^KO^ B6 mice (**d**). **f**, MHC-I peptide selection index showing the frequencies of β5t- and nonβ5t-selected cells among CV CD8^+^ T cells in CD24^−^TCR^+^ mature B6 thymi. **g**, Schematic of CD8^+^ T cell lineage decisions induced by TCR engagements of different thymic peptides in the CD8^Dual^ thymus. CD8^+^ T cell lineage fates are determined by MHC-I TCR signaling duration that is regulated by *Cd4*/*Cd8* co-receptor gene loci and by thymic MHC-I peptides. TCR engagements of β5t-peptides that are expressed exclusively in cTECs in the cortex invariably become disrupted during positive selection, which generates only cytotoxic CD8^+^ T cells. TCR engagements of nonβ5t-peptides that are expressed in the cortex and throughout the thymus might also become disrupted and generate cytotoxic CD8^+^ T cells, but these cells will reencounter nonβ5t-peptides on thymic elements outside the cortex, which will stimulate late TCR signaling that will induce cells, together with thymic IL-4, to become IM CD8^+^ T cells. However, TCR engagements with high affinity of nonβ5t-peptides expressed throughout the thymus might persist without disruption, which leads to the generation of helper CD8^+^ T cells. Numbers within profiles indicate frequency of cells in each box (**a** and **d**). ***P < 0.001, **P < 0.01, *P < 0.05 (two-tailed unpaired *t*-tests); mean ± s.e.m. (**b** and **e**). CMJ, corticomedullary junction.

**Extended Data Fig. 8:**
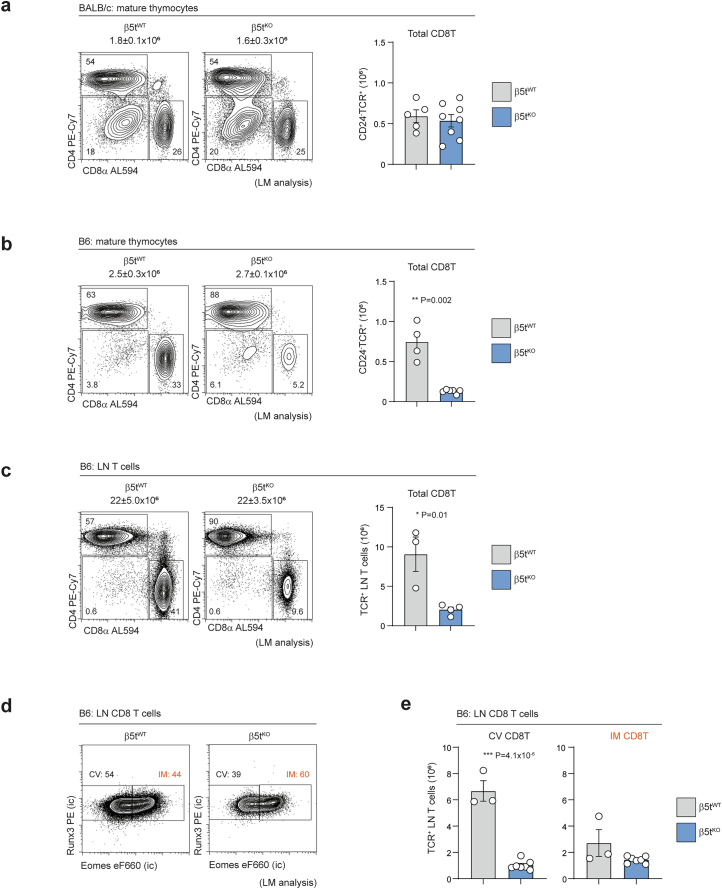
IM CD8 T cells in WT mice also require nonβ5t-peptides. **a**. Flow cytometry of CD24^−^TCR^+^ mature thymocytes and numbers of mature CD8 thymocytes from LM control β5t^WT^ (n = 5) and β5t^KO^ BALB/c (n = 8) mice (representative of 4-5 independent experiments). **b**. Flow cytometry of CD24^−^TCR^+^ mature thymocytes and numbers of mature CD8 thymocytes from LM control β5t^WT^ (n = 4) and β5t^KO^ (n = 6) B6 mice (representative of 4-6 independent experiments). **c**. Flow cytometry of TCR^+^ LN T cells and numbers of CD8 LN T cells from LM control β5t^WT^ (n = 3) and β5t^KO^ (n = 4) B6 mice (representative of 3-4 independent experiments). **d**. Intracellular staining (ic) of Runx3 and Eomes in CD8 T cells among TCR^+^ LN T cells from LM control β5t^WT^ (n = 5) and β5t^KO^ (n = 10) B6 mice (representative of 5-8 independent experiments). **e**. Numbers of CV (Runx3^+^Eomes^−^) and IM (Runx3^+^Eomes^+^) CD8 T cells among TCR^+^ LN T cells from LM control β5t^WT^ and β5t^KO^ B6 mice (d). Numbers within profiles indicate frequency of cells in each box (a-d). ***P < 0.001, **P < 0.01, *P < 0.05 (two-tailed unpaired *t*-tests); mean±s.e.m (a-c,e).

Finally, we wanted to determine if β5t-peptides and nonβ5t-peptides also select CV and IM CD8^+^ T cells in WT BALB/c and B6 mice, as they did in CD8^Dual^ mice. Because WT BALB/c mice resemble CD8^Dual^ mice in containing IL-4-producing NKT2 cells^39,59^, we first examined the impact of thymic peptides on CD8^+^ T cell differentiation in BALB/c thymi. To do so, we bred the β5t^KO^ allele into BALB/c mice and compared CD8^+^ T cells in intact (β5t^WT^) and β5t-deficient (β5t^KO^) BALB/c littermates. Indeed, we discovered that absent β5t-peptides in β5t^KO^ BALB/c mice nearly abrogated generation of CV CD8^+^ T cells but did not significantly affect generation of IM CD8^+^ T cells in the thymus (Fig. 6a–c and Extended Data Fig. 8a). Thus, concordant with our findings in CD8^Dual^ thymi, β5t-peptides select CV CD8^+^ T cells and nonβ5t-peptides mostly select IM CD8^+^ T cells in WT BALB/c mice. Because of the greater number of IM CD8^+^ T cells in BALB/c thymi, which were selected by nonβ5t-peptides, β5t deficiency had little effect on total CD8^+^ T cell number in BALB/c compared to B6 thymi (Extended Data Fig. 8a,b).

**Extended Data Fig. 9:**
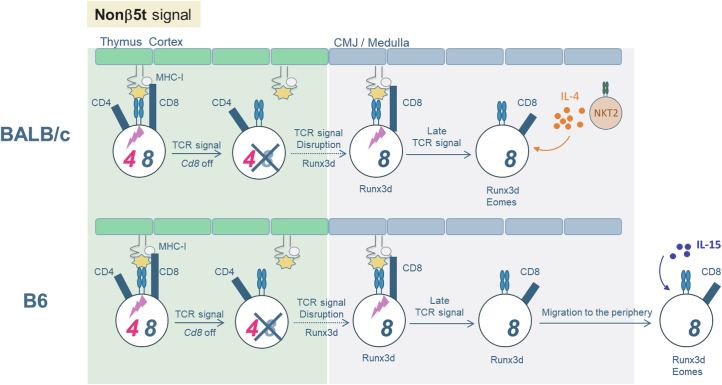
IM CD8 T cell development in B6 and BALB/c WT mice. Nonβ5t-selected cytotoxic CD8 T cells re-encounter nonβ5t-peptides after migrating outside of the cortex and receive late TCR signaling in both BALB/c and B6 thymi. In BALB/c thymi with an abundance of IL-4-producing NKT2 cells, late-TCR signaled CD8 T cells respond to intra-thymic IL-4 which promotes Eomes expression, proliferation, and IM CD8 T cell fate. In contrast, in B6 thymi with few IL-4-producing NKT2 cells, most late-TCR signaled CD8 T cells do not acquire Eomes expression, do not proliferate, and do not acquire IM fate in the thymus. However, after late-TCR signaled CD8 T cells leave the B6 thymus and become peripheral CD8 T cells, we think they encounter IL-15 in the periphery which induces Eomes expression and conversion to IM CD8 T cells.

We then assessed CD8^+^ T cell selection by β5t- and nonβ5t-peptides in B6 thymi, which contain few IL-4-producing NKT2 cells and very few IM CD8^+^ T cells. We found that absent β5t-peptides in β5t^KO^ B6 mice nearly abrogated generation of CV CD8^+^ T cells, revealing that β5t-peptides select CV CD8^+^ T cells in B6 thymi, but that B6 thymi generated too few IM CD8^+^ T cells to allow us to confidently identify their peptide dependence (Fig. 6d–f and Extended Data Fig. 8b). Therefore, we analyzed peripheral CD8^+^ T cells in B6 LNs and found that β5t deficiency significantly reduced the peripheral number of total CD8^+^ and CV CD8^+^ T cells in B6 LNs, but did not reduce the number of IM CD8^+^ T cells in B6 LNs, which are dependent on IL-15 (refs. ^60,65,66^; Extended Data Fig. 8c–e). Consequently, from our assessment of CD8^Dual^, WT BALB/c and WT B6 mice, we conclude that CD8^+^ thymocytes consist mostly of β5t-selected CV T cells and nonβ5t-selected IM T cells, with the exception of WT B6 thymi, which specifically lack IM CD8^+^ T cells because of insufficient thymic IL-4 (Extended Data Fig. 9).

Our perspective on how different thymic peptides impact different CD8^+^ T cell lineage fates in the thymus is schematized in Fig. 6g.

## Discussion

The present study documents that CD8^+^ T cell functionality is determined by MHC-I TCR signaling duration during positive selection and that different MHC-I thymic peptides select functionally different CD8^+^ T cells based on whether the signaling they induce is continuous or disrupted. Analysis of CD8^Dual^ mice reveals that *Cd4*-encoded CD8 co-receptors promote persistent MHC-I TCR signaling, which generates helper CD8^+^ T cells, and that *Cd8*-encoded CD8 co-receptors promote disrupted MHC-I TCR signaling, which generates cytotoxic CD8^+^ T cells. Importantly, CD8^Dual^ mice also reveal that different MHC-I thymic peptides generate functionally different CD8^+^ T cells, in that β5t-peptides (produced by the thymoproteasome) promote only disrupted TCR signaling, which generates cytotoxic CD8^+^ T cells, and that nonβ5t-peptides (produced by other proteasomes) promote persistent or recurrent TCR signaling, which generates helper and IM CD8^+^ T cells. Thus, different MHC-I thymic peptides stimulate generation of distinct subsets of helper, cytotoxic and IM CD8^+^ T cells. Importantly, generation of functionally distinct CD8^+^ T cell subsets by different MHC-I thymic peptides is not unique to CD8^Dual^ mice but is also true in normal WT mice as well.

The present study provides a new perspective on the role of MHC-I thymic selecting peptides in CD8^+^ T cell-positive selection that challenges the current understanding of their role in CD8^+^ T cell generation. It is currently thought that CD8^+^ T cell selection in the thymus is mainly mediated by a specialized subset of MHC-I thymic peptides (referred to as β5t-peptides) that are produced by thymoproteasomes and expressed exclusively in cTECs^57,58^. Other MHC-I thymic peptides (nonβ5t-peptides) are produced by other proteasomes and are expressed throughout the thymus, including in cTECs, but these peptides are thought to be poor selectors of CD8^+^ T cells. Indeed, previous reports performed in B6-background mice have revealed that most CD8^+^ T cells are selected by thymic β5t-peptides and have revealed that β5t-selected CD8^+^ T cells are more TCR responsive and functional than nonβ5t-selected CD8^+^ T cells^67–69^. Consequently, thymoproteasome-generated β5t-peptides are thought to express particular amino acid sequences that make them uniquely able to stimulate MHC-I TCR to signal thymocyte selection and differentiation into functional CD8^+^ T cells^70,71^.

In contrast, our present study now documents that β5t-peptides and nonβ5t-peptides both stimulate CD8^+^ T cell-positive selection but that they generate functionally different CD8^+^ T cells, with β5t-selected CD8^+^ T cells acquiring only cytotoxic function and nonβ5t-selected CD8^+^ T cells becoming either helper and/or IM cells. Because β5t-peptides are expressed only in cTECs, TCR engagement of β5t-peptides would invariably become disrupted when thymocytes disengage from the cortex and invariably become cytotoxic CD8^+^ T cells; and, because nonβ5t-peptides are expressed throughout the thymus, TCR engagement of nonβ5t-peptides would either continue or recur when thymocytes disengage from the cortex and migrate through the thymus while becoming helper or IM CD8^+^ T cells. Thus, it is because MHC-I thymic peptides have different expression patterns in the thymus that they stimulate the generation of functionally different (helper, cytotoxic, IM) CD8^+^ T cells. Nevertheless, because MHC-I TCR clearly distinguish β5t-peptides from nonβ5t-peptides, the present study does not exclude the possibility that β5t-peptides may be intrinsically more stimulatory of MHC-I TCR than nonβ5t-peptides.

It is interesting to consider that the present study suggests that transient termination of *Cd8* gene expression as emphasized in the kinetic signaling model may be unnecessary for β5t-signaled thymocytes to become CD8 cytotoxic T cells because β5t-induced TCR signaling would be disrupted anyway when signaled thymocytes disengage from cTECs and migrate out of the thymic cortex. In contrast, transient termination of *Cd8* gene expression is necessary for nonβ5t-signaled thymocytes to adopt cytotoxic fates. Consequently, while transient termination of *Cd8* gene expression is a universal mechanism for disrupting CD8-dependent MHC-I TCR signaling during positive selection, TCR signaling disruption of positive selection by any mechanism induces cytotoxic fate.

The effect of MHC-I thymic peptides on CD8^+^ T cell lineage fates in the present study was not limited to CD8^Dual^ mice as it was also documented in normal mice, although BALB/c and B6 normal mice differed from one another in terms of thymic IL-4 amounts. BALB/c thymi contain IL-4-producing NKT2 cells and intrathymic IL-4 in amounts that support robust numbers of nonβ5t-selected IM CD8^+^ T cells. In contrast B6 thymi contain few IL-4-producing NKT2 cells and little intrathymic IL-4, as well as containing very few nonβ5t-selected IM CD8^+^ T cells. Thus, we think it is their deficient number of IM CD8^+^ T cells that explains why B6 thymocytes contain mainly β5t-selected CD8^+^ T cells and few nonβ5t-selected CD8^+^ T cells. As possible underlying mechanisms, we think that thymocytes receiving late TCR signaling by nonβ5t-peptides may require thymic IL-4 for survival as well as to become IM CD8^+^ T cells; or, alternatively, it is possible that very few CD8^+^ thymocytes are signaled by nonβ5t-peptides but these then extensively proliferate in response to abundant thymic IL-4. While preliminary experiments suggest that in vivo expression of an antiapoptotic transgene does not reveal additional nonβ5t-signaled thymocytes, we cannot yet definitively distinguish between these two possibilities.

In any event, by revealing that functionally different CD8^+^ T cell subsets are generated by different MHC-I thymic peptides, our study provides new insights into the different developmental requirements for different CD8^+^ T cell subsets. Helper CD8^+^ T cells are only generated by TCR engagement of nonβ5t-peptides, which can persist and continue undisrupted despite thymocyte migration out of the thymus. However, if TCR engagement of nonβ5t-peptides becomes disrupted, they can reengage nonβ5t-peptides on non-cortical thymic elements to stimulate late TCR signaling, which induce thymocytes to become IM CD8^+^ T cells. In contrast to helper and IM CD8^+^ T cells, conventional CD8 cytotoxic T cells are generated by TCR engagement of β5t-peptides, which would become permanently disrupted when TCR signaling terminates thymocyte expression of the chemokine receptor CXCR4, which causes signaled thymocytes to disengage from cTECs and to migrate out of the cortex without receiving additional late TCR signaling^56^. A recent bioinformatics analysis suggested that late TCR signaling may be required for generation of all mature CD8^+^ T cells^3,72,73^. However, our current study documents that late TCR signaling is only required for generation of IM CD8^+^ T cells but is not required for generation of any other CD8^+^ T cells. How late TCR signaling, together with thymic IL-4, generates IM CD8^+^ T cells requires further investigation, but we think that late TCR signaling may prolong the encounter of developing CD8^+^ T cells with thymic IL-4 either by delaying their exit from the thymus or by increasing thymocyte responsiveness to IL-4 in some other way. Notably, our present findings support the concept that IM CD8^+^ T cells are generated by a TCR-instructed process in the thymus that causes IM CD8^+^ T cells to express a different TCR repertoire than conventional CD8^+^ T cells, as has been reported^74^. While it was the abundance of IL-4-producing NKT2 cells in CD8^Dual^ thymi that revealed that IM CD8^+^ T cells required late TCR signaling by nonβ5t-peptides, the reason CD8^Dual^ thymi contain an abundance of IL-4-producing NKT2 cells is not yet understood. Because CD8^Dual^ thymi contain *Cd4*-encoded CD8 co-receptors whose surface expression is maintained throughout positive selection, its basis may be related to the finding that constitutive expression of transgenic CD8 also results in an abundance of IL-4-producing NKT2 cells^75,76^.

Finally, the concept that thymocyte migration affects CD8^+^ T cell lineage fate determination as proposed in the present study merits further comment. The fact that β5t-peptides expressed exclusively by cTECs signal CD8^+^ thymocytes to only become cytotoxic T cells, whereas nonβ5t-peptides expressed throughout the thymus signal thymocytes to instead become helper or IM CD8^+^ T cells provides strong evidence that thymocyte migration contributes to these CD8^+^ T cell lineage fates. Notably, future experiments will directly assess if disruption of thymocyte migration indeed alters CD8^+^ T cell lineage fates.

In conclusion, CD8^+^ T cell lineage fates are determined by MHC-I TCR signaling persistence or disruption during positive selection, with disruption of TCR signaling resulting in generation of cytotoxic CD8^+^ T cells. Concordant with this perspective, the expression pattern of different MHC-I thymic selecting peptides affects CD8^+^ T cell lineage fate decisions by causing persistent or disrupted TCR signaling. Thus, the present study integrates thymocyte peptide specificity with CD8^+^ T cell fate determination during MHC-I signaled positive selection.

## Methods

### Mice

B6 (CD45.1 and CD45.2) mice were purchased from Charles River Laboratory. Aire^KO^ (ref. ^77^), BALB/cJ, β2m^KO^  (ref. ^78^), CD1d^KO^  (ref. ^79^), CD8α^KO^  (ref. ^80^), IL-4R^KO^  (ref. ^81^) and IL-15^KO^  (ref. ^82^) mice were purchased from The Jackson Laboratory. β5t^KO^ from Y.T.^57^, PLZF^KO^ from D. Kovalovsky^35^, MHC-II^KO^, HY.Rag2^KO^, P14.Rag2^KO^, OT-I.Rag2^KO^, Rag-GFP transgene (Tg) from M. Nussenzweig^83^, Runx3d-YFP knock-in from D. R. Littman^84^ and ThPOK-GFP Tg mice from R. Bosselut^25^ were maintained in our own animal colony. To generate BALB/c.β5t^KO^ mice, B6.β5t^KO^ mice were back-crossed five times with BALB/cJ. Mice were housed on a 12-h light–dark cycle at 20–26 °C with 30–70% humidity in accordance with US National Institutes of Health guidelines. All mice were analyzed without randomization or blinding at 6–12 weeks of age and both sexes were used unless mentioned in the paper. All mouse experiments were approved by the National Cancer Institute Animal Care and Use Committee.

### Generation of CD8^Dual^ mice

CD8^Dual^ mice contained altered *Cd4* gene loci encoding CD8α.1 and CD8β proteins (*Cd4*^CD8^) as previously reported^20^, and contained intact *Cd8* gene loci encoding CD8α.2 and CD8β proteins (Fig. 1a).

### Homeostatic proliferation assays

Donor T cells (CD45.2) purified from LNs using the Pan T Cell Isolation Kit II (Miltenyi Biotec) were labeled with 0.5 μM Cell Trace Violet (Invitrogen) and were injected intravenously into host B6 (CD45.1) mice that were sublethally irradiated (600 R) the previous day. Mice were analyzed 7 days after injection.

### Flow cytometry

Single-cell suspensions (1–5 × 10^6^) were first incubated with anti-FcR (clone 2.4G2; 1:250 dilution) and stained with fluorescence-conjugated antibodies at 4 °C for 40 min in HBSS (Thermo Fisher Scientific) with 0.5% BSA and 0.5% NaN_3_. After washing, stained cells were incubated with fluorescence-conjugated streptavidin against biotin-conjugated antibodies at 4 °C for 15 min. Staining of CCR7 (4B12; 1:50 dilution) was performed at 37 °C for 30 min and staining of CD1d tetramer (CD1d/PBS-57; 1:100 dilution) was performed at 4 °C for 30 min before staining with other antibodies. GM1 amount was analyzed by staining with recombinant cholera toxin subunit B (Thermo Fisher Scientific; 1:200 dilution)^32^. For intracellular staining of transcription factors and cytokines, cells were fixed and permeabilized with the Foxp3 Staining Buffer Set (Thermo Fisher Scientific) or BD Cytofix/Cytoperm Fixation/Permeabilization Kit (BD Biosciences), and then stained with fluorescence-conjugated antibodies at 4 °C for 30 min. Gata3 (TWAJ; 5 μl), ThPOK (T43-94, 2 μl), Runx3 (R3-5G4, 5 μl), PLZF (R17-809; 1:50 dilution), RORγt (Q31-378; 1:50 dilution) and T-bet (4B10; 1:100 dilution) were stained at 4 °C for 1 h. For cytokine staining, cells were stimulated at 37 °C with PMA (50 ng ml^−1^; Calbiochem) and ionomycin (1 μg ml^−1^; Calbiochem) for 2 h and added GolgiStop (BD Biosciences) for 2 h before staining. Stained cells were analyzed on a FACS LSR II or FACS Fortessa flow cytometer (BD Biosciences). Electronic cell sorting was performed on a FACSAria II or a FACSAria FUSION (BD Biosciences). Dead cells were excluded by forward light-scatter gating and staining with propidium iodide or LIVE/DEAD Fixable Aqua Dead Cell Stain Kit (Thermo Fisher Scientific) for fresh and fixed staining, respectively. Data were analyzed using FlowJo software (TreeStar). Anti-CD8α.1 (BioXcell; HB129/116-13.1) and CD8α.2 (BioXcell; 2.43) were labeled with biotin by EZ-Link Sulfo-NHS-LC-Biotinylation Kit (Thermo Scientific) or with Alexa 647 (BioXcell). Detailed information on antibodies is provided in the Reporting Summary.

### In vitro stimulation of LN T cells

T cells were purified from LNs with the Pan T cell Isolation Kit (Miltenyi Biotec). For CD40L induction, LN T cells were stimulated with or without plate-bound anti-CD3 (1 μg ml^−1^) and CD28 (1 μg ml^−1^) antibodies at 37 °C for 24 h. For expression of cytotoxic lineage-related factors, sorted naive LN T cells (TCRβ^+^CD62L^+^CD44^−^) were stimulated with plate-bound anti-CD3 (10 μg ml^−1^) and CD28 (5 μg ml^−1^) antibodies for 3 days followed by stimulation with human IL-2 (100 U ml^−1^) at 37 °C for 2 days.

### T_H_2 cell differentiation assay in vitro

Naive (TCRβ^+^CD62L^+^CD44^−^) T cells were electronically sorted from LNs. Sorted T cells (1 × 10^6^) were stimulated with soluble anti-CD3 (2 μg ml^−1^) in the presence of irradiated (2000R) splenocytes (5 × 10^6^) in each well of a flat-bottom 24-well plate (Corning) at 37 °C for 2 days with hIL-2 (200 U ml^−1^), mIL-4 (10 ng ml^−1^) and anti-IFNγ antibody (20 μg ml^−1^). The cultured T cells were further incubated with hIL-4 (hIL-2 (200 U ml^−1^), mIL-4 (10 ng ml^−1^) and anti-IFNγ antibody (20 μg ml^−1^) in each well of a flat-bottom six-well plate (Corning) 37 °C for 3 days.

### RT–qPCR

Total RNA was isolated with the RNeasy Plus Mini Kit (Qiagen) and cDNA was prepared with superscript III First-Strand Synthesis System for RT–PCR kit (Invitrogen). RT–qPCR was done with TaqMan PCR system (Thermo Fisher Scientific) or QuantiTect SYBR Green PCR system (Qiagen). TaqMan probes for Cd40lg (CD40L; Mm00441911_m1), Cd8a (Mm01182107_g1), Cish (Mm01230623_g1), Eomes (Mm01351985_m1), Il-4 (Mm00445259_m1), Nr4a1 (Nur77; Mm01300401_m1), Prf1 (Perforin; Mm00812512_m1), Rpl13a (Mm01612986_gH), Socs1 (Mm00782550_s1), Socs3 (Mm00545913_s1), Tbx21 (T-bet; Mm00450960_m1) and Zbtb7b (ThPOK; Mm00784709_s1), were from Thermo Fisher Scientific. The primer sequences for SYBR green PCR system were as follows: Runx3d forward (5’-GCGACATGGCTTCCAACAGC-3’) and reverse (5’-CTTAGCGCGCTGTTCTCGC-3’); Rpl13a forward (5’-CGAGGCATGCTGCCCCACAA-3’) and reverse (5’-AGCAGGGACCACCATCCGCT-3’). Samples were analyzed on a QuantStudio 6 Flex Real-time PCR System (Applied Biosystems). Gene expression values were normalized to those of Rpl13a expression in the same sample.

### RNA-seq analysis

Naive (TCRβ^+^CD62L^+^CD44^−^) T cells were electronically sorted from LNs. Total RNA was prepared from sorted cells with the RNeasy Plus Mini Kit (Qiagen). The quality of RNA was assessed by Bioanalyzer (Agilent), and RNA samples with an RNA integrity number >9 were used. The library was made by using the SMARTer Universal Low Input RNA Kit (Clontech) for sequencing. The sequencing was performed with a paired-end sequencing length of 125 base pairs by using HiSeq 2500 equipment (Illumina). Reads were aligned to the mouse genome (mm10) with STAR aligner^85^, and raw count data were produced using RSEM^86^. Differentially expressed genes (DEGs) were genes whose fold change was more than 5-fold and *P* value was less than 0.05. DEGs between CD4^+^ and CD8^+^ T cells from B6 mice (631 genes) were analyzed in CD8.1 and CD8.2 T cells from CD8^Dual^ mice. Visualization of DEGs is shown in a heat map generated with Partek.

### Differentiation time of double-positive thymocytes into CV and IM CD8^+^ T cells

To determine the time for CV and IM CD8^+^ T cell development from double-positive thymocytes, Rag-GFP Tg was introduced into CD8^Dual^ mice and Rag-GFP expression (MFI) on CD8^Dual^ thymocytes was analyzed. Rag-GFP expression (MFI) in CV and IM CD8.2 T cells relative to CD24^+^CD8α.1^+^CD8β^+^ double-positive thymocytes were used to calculate the differentiation time based on a Rag-GFP half-life of 54–56 h as: time (h) = (100 – relative Rag-GFP expression)/0.9 (ref. ^62^).

### MHC-I peptide selection index

We calculated the frequencies of β5t-selected and nonβ5t-selected cells based on cell numbers from β5t^WT^ and β5t^KO^ mice in the following way:

$${\rm{Non}}\beta 5{\rm{t}}_{\rm{selected}}\,{\rm{cells}}\;({\rm{ \% }})=({{\rm{No}}.\;\beta 5{\rm{t}}}^{{\rm{KO}}}\!/{{\rm{No}}.\;\beta 5{\rm{t}}}^{{\rm{WT}}})\times 100$$

$$\beta 5{\rm{t}}_{\rm{selected}}\,{\rm{cells}}\;({\rm{ \% }})=[1-({{\rm{No}}.\;\beta 5{\rm{t}}}^{{\rm{KO}}}\!/{{\rm{No}}.\;\beta 5{\rm{t}}}^{{\rm{WT}}})]\times 100$$

Values greater than 100 were set to 100.

### Statistical analysis

Statistical analyses were performed using an unpaired Student’s *t*-test. *P* values < 0.05 were considered significant.

### Reporting summary

Further information on research design is available in the Nature Portfolio Reporting Summary linked to this article.

## Online content

Any methods, additional references, Nature Portfolio reporting summaries, source data, extended data, supplementary information, acknowledgements, peer review information; details of author contributions and competing interests; and statements of data and code availability are available at 10.1038/s41590-025-02411-4.

## Supplementary information

Reporting Summary

## Data Availability

RNA-seq of LN T cells from CD8^Dual^ and B6 mice are deposited in the Gene Expression Omnibus under accession no. GSE297710.
