# Supplementary material for: Unraveling CD8 lineage decisions reveals that functionally distinct CD8+ T cells are selected by different MHC-I thymic peptides
Source: Nat Immunol. 2026 Jan 19;27(4):786–98. doi: 10.1038/s41590-025-02411-4 (PMC13043308; doi:10.1038/s41590-025-02411-4)
Supplement: Supplementary file 1 — Reporting Summary [file 41590_2025_2411_MOESM1_ESM.pdf]

nature portfolio

Corresponding author(s): Alfred Singer

Last updated by author(s): Oct 30, 2025

Reporting Summary

Nature Portfolio wishes to improve the reproducibility of the work that we publish. This form provides structure for consistency and transparency in reporting. For further information on Nature Portfolio policies, see our Editorial Policies and the Editorial Policy Checklist.

Statistics

For all statistical analyses, confirm that the following items are present in the figure legend, table legend, main text, or Methods section.

n/a	Confirmed	
☐	☒	The exact sample size (*n*) for each experimental group/condition, given as a discrete number and unit of measurement
☐	☒	A statement on whether measurements were taken from distinct samples or whether the same sample was measured repeatedly
☐	☒	The statistical test(s) used AND whether they are one- or two-sided<br>*Only common tests should be described solely by name; describe more complex techniques in the Methods section.*
☒	☐	A description of all covariates tested
☐	☒	A description of any assumptions or corrections, such as tests of normality and adjustment for multiple comparisons
☐	☒	A full description of the statistical parameters including central tendency (e.g. means) or other basic estimates (e.g. regression coefficient) AND variation (e.g. standard deviation) or associated estimates of uncertainty (e.g. confidence intervals)
☐	☒	For null hypothesis testing, the test statistic (e.g. *F*, *t*, *r*) with confidence intervals, effect sizes, degrees of freedom and *P* value noted<br>*Give P values as exact values whenever suitable.*
☒	☐	For Bayesian analysis, information on the choice of priors and Markov chain Monte Carlo settings
☒	☐	For hierarchical and complex designs, identification of the appropriate level for tests and full reporting of outcomes
☒	☐	Estimates of effect sizes (e.g. Cohen's *d*, Pearson's *r*), indicating how they were calculated

Our web collection on statistics for biologists contains articles on many of the points above.

Software and code

Policy information about availability of computer code

Data collection	LSRII, Fortessa, FACSAriaII, FACSAria FUSION (BD Biosciences), QuantStudio 6 Flex Real-time PCR System (Applied Biosystems), HiSeq2500 equipment (Illumina).
Data analysis	FlowJo v10.6.2, Prism 10 (Graph pad software), Partek version 7 (Partek Inc).

For manuscripts utilizing custom algorithms or software that are central to the research but not yet described in published literature, software must be made available to editors and reviewers. We strongly encourage code deposition in a community repository (e.g. GitHub). See the Nature Portfolio guidelines for submitting code & software for further information.

Data

Policy information about availability of data

All manuscripts must include a data availability statement. This statement should provide the following information, where applicable:

- Accession codes, unique identifiers, or web links for publicly available datasets
- A description of any restrictions on data availability
- For clinical datasets or third party data, please ensure that the statement adheres to our policy

RNA-sequencing data of LN T cells from B6 and CD8 Dual mice (GEO: GSE297710)

Research involving human participants, their data, or biological material

Policy information about studies with human participants or human data. See also policy information about sex, gender (identity/presentation), and sexual orientation and race, ethnicity and racism.

Reporting on sex and gender	N/A
Reporting on race, ethnicity, or other socially relevant groupings	N/A
Population characteristics	N/A
Recruitment	N/A
Ethics oversight	N/A

Note that full information on the approval of the study protocol must also be provided in the manuscript.

Field-specific reporting

Please select the one below that is the best fit for your research. If you are not sure, read the appropriate sections before making your selection.

☒ Life sciences ☐ Behavioural & social sciences ☐ Ecological, evolutionary & environmental sciences

For a reference copy of the document with all sections, see nature.com/documents/nr-reporting-summary-flat.pdf

Life sciences study design

All studies must disclose on these points even when the disclosure is negative.

Sample size	Sample size for each experiment is indicated in figure legends. At least two independent experiments with three replicates were conduced as the minimum sample size.
Data exclusions	No data were excluded.
Replication	For all experiments, at least three replicates were analyzed in at least two independent experiments. The experimental findings were reliably reproduced.
Randomization	Animals were allocated to groups based on genotype.
Blinding	No blinding was used.

Reporting for specific materials, systems and methods

We require information from authors about some types of materials, experimental systems and methods used in many studies. Here, indicate whether each material, system or method listed is relevant to your study. If you are not sure if a list item applies to your research, read the appropriate section before selecting a response.

Materials & experimental systems

n/a	Involved in the study	
☐	☒	Antibodies
☒	☐	Eukaryotic cell lines
☒	☐	Palaeontology and archaeology
☐	☒	Animals and other organisms
☒	☐	Clinical data
☒	☐	Dual use research of concern
☒	☐	Plants

Methods

n/a	Involved in the study	
☒	☐	ChIP-seq
☐	☒	Flow cytometry
☒	☐	MRI-based neuroimaging

Antibodies

Antibodies used	Antibody, supplier, catalogue #, clone #.<br><br>2.4G2 Harlan G208312 2.4G2

CCR7 Biotin Thermo Fisher Scientific 13-1971-85 4B12
CCR7 PE Thermo Fisher Scientific 12-1971-83 4B12
CD103 PE Thermo Fisher Scientific 12-1031-83 Clone: 2E7
CD122 PE BD Biosciences 553362 TM-beta 1
CD24 eF780 Thermo Fisher Scientific 47-0242-82 M1/69
CD25 PE BD Pharmingen 553866 PC61
CD28 BD pharmingen 553294 37.51
CD28 PE Thermo Fisher Scientific 12-0281-82 37.51
CD3 BD pharmingen 553057 145-2C11
CD4 AL594 Biolegend 100446 GK1.5
CD4 PE-Cy7 Thermo Fisher Scientific 25-0042-82 RM4-5
CD40L PE Thermo Fisher Scientific 12-1541-82 MR1
CD44 APC Biolegend 103012 IM7
CD45.2 PE-Cy7 Biolegend 109830 104
CD49d PE Biolegend 103608 R1-2
CD5 Pacific Blue Biolegend 100642 53-7.3
CD5 PE BD Pharmingen 553023 53-7.3
CD69 APC Biolegend 104514 H1.2F3
CD69 Biotin BD Pharmingen 553235 H1.2F3
CD69 BV786 BD Biosciences 564683 H1.2F3
CD69 PE BD Pharmingen 553237 H1.2F3
CD69 PE-Cy7 Biolegend 104512 H1.2F3
CD8a AL594 Biolegend 100758 53-6.7
CD8a.1 BioXcell BE0118 HB129/116-13.1
CD8a.2 BioXcell BE0061 2.43
CD8a.2 APC TONBO biosciences 20-1886-U100 2.43
CD8a.2 PE-Cy7 TONBO biosciences 60-1886-U100 2.43
CD8b Pacific blue Biolegend 140414 53.5.8
CXCR3 PE Biolegend 126506 CXCR3-173
Eomes eF660 Thermo Fisher Scientific 50-4875-82 Dan11mag
Foxp3 eF660 Thermo Fisher Scientific 50-5773-82 FJK-16s
Gata3 PE Thermo Fisher Scientific 47-0042-82 TWAJ
Granzyme B AL647 Biolegend 515406 GB11
HY TCR FITC Thermo Fisher Scientific 11-9930-82 T3.70
IFN-g PE Biolegend 505808 XMG1.2
IL-17 APC Thermo Fisher Scientific 17-7177-81 eBio17B7
IL-4 APC Biolegend 504106 11B11
IL-7R PE eBioscience 12-1271-82 A7R34
Ly6C BV786 BD Biosciences 569011 AL-21
PLZF AL647 BD Biosciences 563490 R17-809
PLZF PE Biolegend 145804 Clone: 9E12
Qa-2 AL647 Biolegend 121708 695H1-9-9
RORgt BV421 BD Biosciences 562894 Q31-378
Runx3 PE BD Biosciences 564814 R3-5G4
Streptavidin AL594 Thermo Fisher Scientific S11227
T-bet eF660 Thermo Fisher Scientific 50-5825-82 4B10
TCRb AL647 Life Technologies HM3621 H57-597
TCRb FITC BD Pharmingen 553171 H57-597
ThPOK AL647 BD Biosciences 565500 T43-94
Va2 FITC Thermo Fisher Scientific 11-5812-82 B20.1

Validation

All antibodies are commercially available and have been validated by the manufactures.

Animals and other research organisms

Policy information about studies involving animals; ARRIVE guidelines recommended for reporting animal research, and Sex and Gender in Research

Laboratory animals

Mouse strain, source, catalogue #

CD45.1 B6 Charles River Laboratory #564
CD45.2 B6 Charles River Laboratory #027
AireKO The Jackson Laboratory #36465
BALB/cJ The Jackson Laboratory #651
b2mKO The Jackson Laboratory #2087
CD1dKO The Jackson Laboratory #3814
CD8aKO The Jackson Laboratory #2665
IL-4RKO The Jackson Laboratory #3514
IL-15KO The Jackson Laboratory #34239
B6. b5tKO Murata S et al., 2007
PLZFKO Kovalovsky D et al., 2008
Rag-GFP Yu et al., 1999
Runx3d-YFP knock-in Egawa T et al., 2008
ThPOK-GFP knock-in Wang L et al., 2008

	CD8Dual Shinzawa M et al., 2022<br>B6. b5tKO mice were back-croseed five times with BALB/cJ mice.<br>MHC-IIKO, HY.Rag2KO, P14-Rag2KO, OT-I. Rag2KO mice were maintained in our own animal colony.
Wild animals	N/A
Reporting on sex	Both male and female mice were used and analyzed at age 6-10 weeks old.
Field-collected samples	N/A
Ethics oversight	All animal experiments were approved by the National Cancer Institute Animal Care and Use Committee and were maintained in accordance with US National Institutes of Health guidelines.

Note that full information on the approval of the study protocol must also be provided in the manuscript.

Plants

Seed stocks	N/A
Novel plant genotypes	N/A
Authentication	N/A

Flow Cytometry

Plots

Confirm that:

☒ The axis labels state the marker and fluorochrome used (e.g. CD4-FITC).

☒ The axis scales are clearly visible. Include numbers along axes only for bottom left plot of group (a 'group' is an analysis of identical markers).

☒ All plots are contour plots with outliers or pseudocolor plots.

☒ A numerical value for number of cells or percentage (with statistics) is provided.

Methodology

Sample preparation	Single cell suspensions were prepared in cold HBSS supplemented with 0.5% BSA and 0.5% NaN3.
Instrument	LSRII, Fortessa, FACSAriaII, FACSAria FUSION (BD Biosciences).
Software	FlowJo v10.6.2
Cell population abundance	More than 95% on sorted cells, which was determined by flow cytometry analysis on post sorted cells.
Gating strategy	Live cells were defined by FSC gating and staining with propidium iodide or LIVE/DEAD Fixable Aqua Dead Cell Stain Kit (Thermo Fisher Scientific) for fresh and fixed staining, respectively. All gating strategies are stated in the manuscript.

☒ Tick this box to confirm that a figure exemplifying the gating strategy is provided in the Supplementary Information.
